# Levels and Effects of Nogo‐B in Patients With Type 2 Diabetes or Hyperglycemic HUVEC Model

**DOI:** 10.1002/edm2.70188

**Published:** 2026-03-03

**Authors:** Laurent Irakoze, Linqiang Ma, Yuanfeng Gu, Xiangjun Chen, Fanling Zeng, Rong Luo, Yulian Lai, Xun Li, Shangbin Chen, Paul Banderembako, Liliane Nkengurutse, Xun Lei, Xiaoqiu Xiao, Qingfeng Cheng

**Affiliations:** ^1^ Department of Endocrinology The First Affiliated Hospital of Chongqing Medical University Chongqing China; ^2^ Roi Khaled Hospital Bujumbura Burundi; ^3^ The Chongqing Key Laboratory of Translational Medicine in Major Metabolic Diseases Chongqing China; ^4^ Physical Examination Center, the First Affiliated Hospital of Chongqing Medical University Chongqing China; ^5^ Ministry of Public Health and Fighting AIDS, Epidemiological Emergency Service Bujumbura Burundi; ^6^ School of Public Health Chongqing Medical University Chongqing China

**Keywords:** endothelial‐to‐mesenchymal transition, Nogo‐B, type 2 diabetes, vasculopathy

## Abstract

**Background:**

There is still a lack of enough evidence about Nogo‐B levels and vascular complications in patients with type 2 diabetes. Our first aim was to assess the levels of Nogo‐B in type 2 diabetes mellitus (T2DM) patients with or without vascular complications (VC). Our second aim was to determine the mechanism by which Nogo‐B may protect vasculature using a hyperglycemic HUVEC model.

**Methods:**

Sera or samples of patients with T2DM and subjects without diabetes were collected from the First or Second Affiliated Hospital of Chongqing Medical University. Human umbilical endothelial cells (HUVECs) were purchased and treated with high glucose (HG) and/or cholesterol (C) before and after Nogo‐B knockdown or overexpression. Graphpad and SPSS version 27 software were used for statistical analyses.

**Results:**

T2DM patients with vascular complications (DM + VC) displayed significantly lower levels of Nogo‐B when compared with T2DM patients without VC (DM) or subjects without diabetes (NC) (*p <* 0.001). In addition, lower levels of Nogo‐B were independently associated with diabetes and/or VC in T2DM patients. Nogo‐B overexpression reduced the expression of mesenchymal markers (α‐SMA and Collagen‐1), TGF‐β1 and P‐smad2/3, while increasing the expression of endothelial markers (CD31, eNOS and VWF) in HUVECs treated with HG and/or C.

**Conclusion:**

Our study has proved that lower levels of Nogo‐B are independently associated with VC in T2DM patients. In an in vitro model, Nogo‐B alleviates endothelial cell injury by affecting TGF‐β signalling. Further studies are still needed to support or verify our findings.

## Graphical abstract

Nogo‐B levels significantly decrease in T2DM patients with or without vascular complications (VC). Nogo‐B alleviates endothelial cell injury by affecting TGF‐β signalling in an in vitro model.

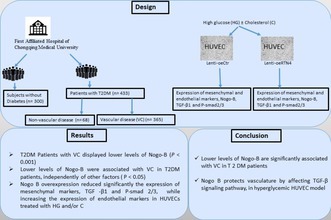

## 1 Introduction

Type 2 Diabetes Mellitus (T2DM) is a chronic metabolic disease, characterised by elevated blood glucose levels [1]. It has been recognised as one of the major risk factors for cardiovascular diseases [2]. In 2021, the prevalence of T2DM was 536.6 million, and it is estimated that this prevalence will reach 783.2 million people globally in 2045 [2].

Chronic hyperglycemia can lead to macrovascular and microvascular complications [3]. The macrovascular complications consist of peripheral artery disease (PAD), cardiovascular diseases and cerebrovascular diseases [3, 4]. The microvascular complications include microvascular diseases such as diabetic nephropathy, diabetic retinopathy and diabetic neuropathy [3, 4]. Some researchers previously concluded that both microvascular and macrovascular complications develop simultaneously in diabetes [5]. Furthermore, the presence of a microvascular disease is also a marker of diffuse vascular disease [4]. It is well known that vascular complications are the leading cause of death among diabetic patients [6]. Due to a lack of early, precise diagnostic markers, many patients with diabetes do not receive early diagnosis and treatment of cardiovascular complications [6]. In addition, many symptomatic treatments for vascular complications are expensive and have unstable therapeutic effects [6]. Therefore, it is important to find accurate diagnostic markers for early detection of vascular complications in diabetes [6].

Nogo‐B, also known as reticulon‐4B (RTN‐4B), is a member of the reticulon protein family [7]. It is mainly localised in the endoplasmic reticulum (ER), where it participates in the maintenance of ER tubular shape and functions [7]. Nogo‐B (RTN‐4B) was previously reported to be widely expressed in vascular cells and cardiomyocytes in vivo and has various cell types in vitro [8]. It is specifically found in the endothelial and smooth muscle cells within the vasculature [9]. Nogo‐B has been shown to be associated with diverse processes such as vascular remodelling, liver regeneration and liver cirrhosis [7].

Many studies have been recently conducted on the association between circulating Nogo‐B levels and some degenerative and/or metabolic disorders [10, 11]. In fact, Hernandez‐Diaz et al. [9] have recently provided the first evidence that a primary increase of sNogo‐B in the circulation protects the glomerular vasculature in diabetes. The same authors finally concluded that Nogo‐B protects the vasculature in diabetes and may represent a novel therapeutic target for diabetic vascular complications. On the contrary, Yang et al. [12] found that Nogo‐B levels were increased in plasma of patients with diabetic retinopathy (DR). They concluded that increased Nogo‐B is an inducer for the retinal vascular permeability, and thus Nogo‐B depletion might be a perspective strategy for prevention and treatment of DR. On the other hand, the current studies on Nogo‐B in vascular diseases of diabetes were mostly conducted in models of type 1 diabetes (in vivo and/or in vitro), with small sample sizes in humans [9, 12, 13]. Therefore, the first aim of our study was to assess whether there is a significant association between levels of Nogo‐B and vascular complications in patients with type 2 diabetes. We hypothesised that levels of Nogo‐B decrease in T2DM patients with or without vascular complications.

Endothelial to mesenchymal transition (EndMT) refers to the process by which normal endothelial cells completely or partially lose their endothelial identity, characterised by expression of certain endothelial markers such as CD31, VE‐cadherin, VWF and acquire expression of mesenchymal markers such as α‐smooth muscle actin, SM22α, calponin and collagens among many others [14, 15, 16]. Previous findings have shown that endothelial dysfunction plays an important role in vascular disease caused by diabetes mellitus [16, 17]. They have also shown that the TGF‐β signalling pathway plays a central role during the process of EndMT [16]. Possible treatment strategies may target the TGF‐β signalling pathways [17].

Human umbilical vein endothelial cells (HUVECs) are a promising candidate for the in vitro study of hyperglycemia‐induced vascular complications [18]. In fact, several studies have used HUVECs to assess the mechanism by which high glucose has detrimental effects on vascular endothelial cells [19, 20]. A recent study suggested that further studies should assess the mechanism by which Nogo‐B protects vasculature in diabetes [9]. We therefore hypothesised that Nogo‐B alleviates endothelial cell injury by inhibiting TGF‐B signalling pathway in hyperglycemic HUVEC model. To the best of our knowledge, this was the first study on this topic.

## 2 Methods

### 2.1 Study Subjects

This study was a comparative single‐centre research study. The Ethics Committee of the First Affiliated Hospital of Chongqing Medical University (China) approved the study (Number: K2023‐537). The experimental procedures were conducted according to the Declaration of Helsinki. As our study was retrospective, we obtained an exemption from the informed consent after contacting the institutional review board (IRB). Additionally, the primary data of the participants were anonymised. Therefore, the medical records of T2DM patients with or without vascular complications were electronically collected from the Department of Endocrinology and Metabolic Diseases, First Affiliated Hospital of Chongqing Medical University. The enrollment period was between January 2021 and November 2024. All patients were confirmed to be diagnosed with T2DM based on the American Diabetes Association (ADA) criteria [21, 22]. Therefore, a fasting plasma glucose (FPG) ≥ 7.0 mmol/L, or haemoglobin A1C (HbA1c) ≥ 6.5% or oral glucose tolerance test (OGGT) 2 h post‐load plasma glucose ≥ 11.1 mmol/L could confirm the diagnosis [16, 21]. In addition, the self‐reported medical history of T2DM could also confirm the diagnosis [16].

T2DM patients with macrovascular complications were those patients with atherosclerotic vascular disease [23, 24]. Those were diseases such as peripheral artery disease (PAD) (history of peripheral artery disease including revascularisation procedures, diabetic foot and amputation), coronary artery disease (CAD) (history of coronary artery disease, angina, myocardial infarction, percutaneous coronary intervention and coronary artery bypass grafting) and cerebrovascular diseases (CVD) (stroke, transient ischemic attack, carotid artery stenting and carotid endarterectomy) [24, 25]. T2DM patients with microvascular complications were those with microangiopathies such as nephropathy or chronic kidney disease (CKD), retinopathy and distal peripheral neuropathy (DPN) [24, 25].

After, medical records of subjects without diabetes mellitus were electronically collected as a control group from the Department of Physical Examination Center, First Affiliated Hospital of Chongqing Medical University. The primary data of participants were also anonymised. All subjects without diabetes consist of volunteers who underwent medical checkup at our institution.

In order to deeply evaluate the association between circulating levels of Nogo‐B and vascular complications in T2DM patients, the included participants were divided into three groups as follows: (a) T2DM patients with vascular complications (DM + VC, *n* = 365), (b) T2DM patients without vascular complications (DM, *n* = 68), (c) subjects without diabetes mellitus (NC, *n* = 300).

In all the groups, the exclusion criteria were as follows: (1) Patients or subjects aged ≥ 80 years or < 40 years, (2) patients with type 1 diabetes mellitus, (3) patients with tumours, (4) patients with liver failure or decompensated liver cirrhosis, (5) patients with respiratory failure, (6) patients with advanced chronic kidney disease (Stage 5), (7) patients with congestive heart failure (NYHA functional classification: class IV), (8) patients with Parkinson disease and (9) patients in any other critical condition such as sepsis.

#### 2.1.1 Diagnosis of Microvascular Complications

A physician guided by available laboratory test reports confirmed the microvascular disease in T2DM patients according to ADA guidelines [26]. Nephropathy and/or CKD in T2DM patients was confirmed in case that there was a decreased estimated glomerular filtration rate (eGFR < 60 mL/min/1.73 m^2^) and/or increased urinary albumin excretion (≥ 30 mg/g creatinine) persisting for > 3–6 months [25, 27]. DR or T2DM with retinopathy was confirmed by an ophthalmologist and/or endocrinologist based on clinical expertise and available techniques such as ophthalmoscopy [27]. 10 g monofilament at four sites on each foot and one of the following: vibration using 128 Hz tuning fork, pinprick sensation, ankle reflexes, and vibration perception threshold were used to diagnose distal peripheral neuropathy (DPN) [28].

#### 2.1.2 Diagnosis of Macrovascular Complications

Physicians and/or clinicians screened for a macrovascular complication in T2DM patients. They referred to standard criteria of the American Heart Association or European Society of Cardiology [16, 29]. The lower extremity peripheral arterial disease (PAD) in T2DM patients was confirmed using techniques such as Ankle‐Brachial Index (ABI) ≤ 0.9 and/or imaging methods such as duplex ultrasound, computer tomography angiography (CTA) or magnetic resonance imaging (MRI) [29]. When necessary, electrocardiogram (ECG), echocardiography, duplex ultrasound, MRI and/or computer tomography coronary angiography (CTCA) were also performed to diagnose other macrovascular diseases such as coronary artery disease (CAD), cerebrovascular disease (CVD), as recommended by guidelines [29].

### 2.2 Clinical Data Collection and Definitions

Demographic characteristics and medical history including health condition and medication were collected through medical records. Cigarette smoking was defined as past or current smoking. Heavy alcohol intake (current/past) was defined as taking an amount of alcohol > 30 g/day in men and > 20 g/day in women [30]. Fasting peripheral venous‐blood samples were collected to obtain the laboratory data, including glycaemia, HbA1c, total cholesterol (TC), low‐density lipoprotein cholesterol (LDL‐C), creatinine, uric acid, triglyceride (TG) and high‐density lipoprotein cholesterol (HDL‐C). Physical examination included measurements of height (m), weight (kg) and body mass index (BMI). BMI was calculated as weight in kg divided by the square of height in metres (kg/m^2^). Hypertension was diagnosed based on a self‐reported physician diagnosis, recent use of an antihypertensive agent, or a BP ≥ 140/90 mmHg [31].

### 2.3 Measurement of Serum Nogo‐B

All recruited participants' venous blood samples were collected with procoagulant vacuum tubes on the morning of the patients' visit after an overnight fasting as previously reported [11]. Blood samples were centrifuged at 3000 g for 15 min at 4°C, and then aliquots of serum were frozen at −80°C until analysis. Human Nogo‐B ELISA Kit (Jiubang Biotechnology Cat# QZ‐17338, RRID: https://www.antibodyregistry.org/AB_3668927) was used to determine serum Nogo‐B levels in all the samples.

## 3 Immunohistochemistry (IHC)

Non‐infected subcutaneous tissues (dermis) were taken from the feet of five patients with diabetic foot ulcers (DFU) in the surgery department of the Second Affiliated Hospital of the Chongqing Medical University. The subcutaneous tissues from the feet of five patients without diabetes (NC) were also taken from the same department. Patients with metabolic disorders other than type 2 diabetes were excluded before matching. All the patients without diabetes consist of patients with foot lesions other than DFU. The term DFU has been defined as a break in the skin of the foot of a person with diabetes, which penetrates as a minimum to the epidermis and part of the dermis [32]. All the participants were matched by sex and age in the two groups. The IRB approved the study (Number: 2020–239). The informed consent was obtained from the participants before experiments. The specimens were washed with cold phosphate buffered saline (PBS) and fixed with 4% paraformaldehyde overnight. They were stored in PBS at 4°C until further analysis. After that, immunohistochemistry was performed as previously reported [33]. In brief, paraffin sections were deparaffinised by putting them into xylene for 15 min, and this step was repeated three times. The sections were rehydrated by sequentially incubating them with 100%, 85% and 75% ethanol for 5 min each. Antigen retrieval was performed using citrate buffer. The slides were incubated with 3% H_2_O_2_ solution for 10 min to quench endogenous peroxidase activity and washed three times with PBS for 5 min each. 3% bovine serum albumin (BSA) was used as blocking serum and the sections were blocked for 30 min. An overnight anti‐Nogo‐B (Abcam Cat# ab47085, RRID: https://www.antibodyregistry.org/AB_881718) was used as primary antibody. Next, the incubation with the corresponding secondary antibody (Affinity Biosciences Cat# S0001, RRID: https://www.antibodyregistry.org/AB_2839429) was performed for 30 min. After that, the DAB chromogenic solution was prepared and used for chromogenesis. After dehydration with alcohol and xylene, the slices were slightly dried, sealed with neutral gum and observed under a microscope.

## 4 Cell Culture

HUVECs were purchased from BeNa Culture Collection (BNCC363216). They were subsequently cultured in HUVEC specific endothelial growth medium (CM‐0122, Procell) containing Ham's F‐12K, 0.1 mg/mL Heparin, 0.03–0.05 mg/mL of endothelial cell growth supplements (ECGs), 10% of foetal bovine serum (FBS) and 1% Penicillin‐Streptomycin (P/S). Before all the experiments, the cells were starved in a serum‐free Ham's F‐F‐12K (21127022, GIBCO) medium for 18–24 h containing antibiotics (1% P/S). All cells used for experiments were with passages 3–9. Additionally, all our experiments were repeated at least three times.

### 4.1 Cell Viability

Cells were seeded (2.10^3^ cells/well) into 96‐well flat‐bottomed plates with three replicates in each group for 24 h. After starvation of the cells, a new fresh medium containing Ham's F‐12k, 10% FBS and 1% P/S was prepared. This medium was subsequently mixed with different amounts of glucose (G8270‐100G, sigma) and/or cholesterol (C3045, sigma). The final concentrations were as follows: (a) 25 mmol/L of glucose (G25); (b) 25 mmol/L of glucose + 100 μmol/L of cholesterol (G25 + C); (c) 33 mmol/L of glucose (G33); (d) 33 mmol/L of Glucose + 100 μmol/L of Cholesterol (G33 + C); (e) 40 mmol/L of glucose (G40); (f) 40 mmol/L of glucose + 100 μmol/L of cholesterol (G40 + C); (g) Cholesterol 100 μmol/L (C). The control group (NC or G7) was a glucose concentration of 7 mmol/L (the standard glucose concentration of Ham's F‐12k). The old medium was then removed, and cells were rinsed twice with PBS. The new fresh medium with the aforementioned treatments was therefore added in the different groups accordingly. CKK‐8 assay kit (GK10001, Glpbio Technology) was used to analyse the cell viability after 24 h and/or 48 h according to the manufacturer's instructions. A 25 mmol/L of glucose concentration was therefore used as an appropriate model of high glucose concentration in the subsequent experiments since it had similar cell viability as 33 mmol/L or 40 mmol/L. Mannitol (M1902‐500G, SIGMA) with a final concentration of 25 mmol/L (M25) was used in order to control the osmotic pressure that might be exerted by glucose. As Cholesterol is not water soluble, it was diluted in ethyl alcohol and the same amount of alcohol was added in the different treatment groups.

### 4.2 Culture and Treatment of Cells in Six‐Well Plates

HUVECs were seeded at a density of 3.10^5^ cells per well in 6‐well culture plates. After, they were incubated at 37°C in 5% CO_2_ for 24 h to create confluent monolayers. After serum starvation for 18 h, the cells were treated with G25, G25 + C and C for 24 h and/or 48 h. The control group was with a final glucose concentration of 7 mmol/L (NC or G7). The cell culture supernatant was collected, aliquoted and frozen at −80°C until further analysis.

### 4.3 Enzyme‐Linked Immunosorbent Assay (ELISA)

ELISA was performed to analyse the expression of inflammatory cytokines (IL‐1, IL‐6 and TNF‐α), TGF‐β1, IL‐10 and Nogo‐B in the cell culture supernatant collected after glucose and/or cholesterol treatments. The kits used were purchased from Quanzhou Jiubang Biotechnology Co. Ltd. (Fujian, China). Those kits were as follows: (1) Human Nogo‐B ELISA Kit (Jiubang Biotechnology Cat# QZ‐17338, RRID: https://www.antibodyregistry.org/AB_3668927), (2) Human IL‐1 ELISA Kit (Jiubang Biotechnology Cat# QZ‐10456, RRID: https://www.antibodyregistry.org/AB_3668928), (3) Human IL‐6 ELISA Kit (Jiubang Biotechnology Cat# QZ‐10469, RRID: https://www.antibodyregistry.org/AB_3668929), (4) Human IL‐10 ELISA Kit (Jiubang Biotechnology Cat# QZ‐12319, RRID: https://www.antibodyregistry.org/AB_3668930), (5) Human TNF‐α ELISA Kit (Jiubang Biotechnology Cat# QZ‐10789, RRID: https://www.antibodyregistry.org/AB_3668931), (6) Human TGF‐β1 ELISA Kit (Jiubang Biotechnology Cat# QZ‐10798, RRID: https://www.antibodyregistry.org/AB_3668932).

### 4.4 Protein Extraction and Western Blotting (WB)

Cells were lysed using RIPA buffer (R0010, Solarbio), proteinase and phosphatase inhibitors (P1045, Beyotime). Protein concentrations were quantified using a bicinchoninic acid assay kit (P0009, Beyotime). Proteins were separated via 10% SDS‐PAGE and transferred onto PVDF membranes. Following the blocking with non‐fat milk (GC310001‐100 g, Servicebio) for 2 h at room temperature, the membranes were incubated at 4°C overnight with primary antibodies. Those antibodies were as follows: anti‐Nogo‐B (Abcam Cat# ab47085, RRID: https://www.antibodyregistry.org/AB_881718), anti‐CD31 (Affinity Biosciences Cat# AF0077, RRID: https://www.antibodyregistry.org/AB_2833268), anti‐α‐SMA (Affinity Biosciences Cat# AF1032, RRID: https://www.antibodyregistry.org/AB_2835329), anti‐Collagen‐1 (Affinity Biosciences Cat# AF7001, RRID: https://www.antibodyregistry.org/AB_2835309), anti‐eNOS (Affinity Biosciences Cat# AF0096, RRID: https://www.antibodyregistry.org/AB_2833277), anti‐VWF (Affinity Biosciences Cat# AF3000, RRID: https://www.antibodyregistry.org/AB_2837580), anti‐Smad2/3 (Cell Signalling Technology Cat# 8685, RRID: https://www.antibodyregistry.org/AB_10889933), anti‐p‐Smad2/3 (Cell Signalling Technology Cat# 8828, RRID: https://www.antibodyregistry.org/AB_2631089) and anti‐TGF Beta‐1 (Thermo Fisher Scientific Cat# MA5‐16949, RRID: https://www.antibodyregistry.org/AB_2538424). Anti‐Beta‐actin (Affinity Biosciences Cat# AF7018, RRID: https://www.antibodyregistry.org/AB_2839420) was used as a control antibody. The secondary antibodies that were used were Goat Anti‐Rabbit (Affinity Biosciences Cat# S0001, RRID: https://www.antibodyregistry.org/AB_2839429) and Goat Anti‐Mouse (Boster Biological Technology Cat# BA1050, RRID: https://www.antibodyregistry.org/AB_2904507).

### 4.5 RNA Extraction and Quantitative Real‐Time PCR (qPCR)

Total RNA was isolated from cells using RNAex reagent (Accurate Biotechnology (Hunan) Co. Ltd., China). Relative quantification by real‐time PCR involves the use of a CFX96 real‐time PCR system (Bio‐Rad Laboratories, USA). Other reagents involved Evo M‐MLVMix Kit with gDNA Clean for qPCR (Accurate Biotechnology (Hunan) Co. Ltd., China) and SYBRGreen PremixPro TaqHS qPCR Kit (Accurate Biotechnology (Hunan) Co. Ltd., China) for real‐time detection of PCR products. The $2^{-\Delta \Delta C_{t}}$ method was used to analyse the relative expression levels. The reaction was performed in triplicate; the reactants were heated to 95°C for 30 s, and then they were cycled 40 times at 95°C for 5 s and heated at 60°C for 30 s. β‐Actin (ACTB) was used as a control gene.

The primers were as follows: RTN4 forward GTTGACCTCCTGTACTGGAGA, RTN4 reverse CTGTTACGCTCACAATGCTGA; PECAM1 forward CCAAGGTGGGATCGTGAGG, PECAM1 reverse TCGGAAGGATAAAACGCGGTC; COL1A1 forward GTGCGATGACGTGATCTGTGA, COL1A1 reverse CGGTGGTTTCTCTTGGGGT; ACTA2 forward AAAAGACAGCTACGTGGGTGA, ACTA2 reverse GCCATGTTCTATCGGGTACTTC; NOS3 forward TGATGGCGAAGCGAGTGAAG, NOS3 reverse ACTCATCCATACACAGGACCC; VWF forward CCTTGACCTCGGACCCTTATG, VWF reverse GATGCCCGTTCACACCACT; β‐Actin forward CATGTACGTTGCTATCCAGGC, β‐Actin reverse CTCCTTAATGTCACGCACGAT; TGFB1 forward GCCGACTACTACGCCAAGGA, TGFB1 reverse ATGCTGTGTGTACTCTGCTTGAAC.

### 4.6 HUVEC Transfection

Vehicle lentivirus (poSLenti‐EF1‐EGFP‐P2A‐Puro‐CMV‐MCS‐3xFLAG‐WPRE or Lenti‐oeCtr) and RTN4 overexpressing lentivirus [poSLenti‐EF1‐EGFP‐P2A‐Puro‐CMV‐RTN4 (Nogo‐1)‐3xFLAG‐WPRE or Lenti‐oeRTN4] were purchased from OBIO Technology (Shanghai, China). Simultaneously, vehicle lentivirus [pSLenti‐U6‐shRNA (NC2)‐CMV‐EGFP‐F2A‐Puro‐WPRE or Lenti‐shCtr] and short hairpin RNA lentivirus (shRNA) targeting RTN4 [pSLenti‐U6‐shRNA (RTN4)‐CMV‐EGFP‐F2A‐Puro‐WPRE or Lenti‐shRTN4] were purchased from the same company. RTN4 silencing target sequence was GCAGTGTTGATGTGGGTATTT (Lenti‐shRTN4‐2) or GCTATATCTGAGGAGTTGGTT (Lenti‐shRTN4‐3). RTN4 silencing control sequence was CCTAAGGTTAAGTCGCCCTCG (lenti‐shCtr). After, the cells were transduced with a multiplicity of infectivity (MOI) of 10 and polybrene 1 mg/mL as described by the manufacturer. The stably infected cells were selected by puromycin. The knockdown or overexpression of RTN4 was confirmed by western blotting and/or PCR. After transfection, the cells were treated with glucose and/or cholesterol as already described. For RTN4 silencing or vehicle lentivirus, the groups formed after treatments were as follows: (1) vehicle lentivirus + G7 (Lenti‐shCtr+G7), (2) vehicle lentivirus + G25 (Lenti‐shCtr+G25), (3) vehicle lentivirus + G25 + C (Lenti‐shCtr+G25 + C), (4) shRNA lentivirus targeting RTN4 + G7 (Lenti‐shRTN4 + G7), (5) shRNA lentivirus targeting RTN4 + G25 (Lenti‐shRTN4 + G25), (6) shRNA lentivirus targeting RTN4 + G25 + C (Lenti‐shRTN4 + G25 + C). For vehicle or RTN4 overexpressing lentivirus, the groups obtained after treatments were as follows: (1) vehicle lentivirus + G7 (Lenti‐oeCtr+G7), (2) vehicle lentivirus + G25 (Lenti‐oeCtr+G25), (3) vehicle lentivirus + G25 + C (Lenti‐oeCtr+G25 + C), (4) RTN4 overexpressing lentivirus + G7 (Lenti‐oeRTN4 + G7), (5) RTN4 overexpressing lentivirus + G25 (Lenti‐oeRTN4 + G25), (6) RTN4 overexpressing lentivirus + G25 + C (Lenti‐oeRTN4 + G25 + C). Subsequently, RNA and proteins were extracted and, therefore, WB and/or quantitative PCR were performed. We finally analysed the expression of mesenchymal markers, endothelial markers, TGF‐B1 (*TGFB1*), P‐smad2/3, smad2/3 and Nogo‐B (*RTN4*). Additionally, the cell culture supernatant was also collected and ELISA was performed to analyse the expression of the aforementioned markers.

### 4.7 Wound Healing Assay

The scratch wound assay was performed as described previously [34, 35]. HUVECs were seeded at a density of 3.10^5^ cells per well in 6‐well culture plates and incubated at 37°C in 5% CO_2_ for 24 h to create confluent monolayers. After serum starvation, the monolayer cells were scratched in a straight line across the center of the plate bottom using a 200‐μL pipette tip. The detached cells were removed by washing with serum‐free medium. The cells were subsequently treated with high glucose (G25) and/or C as already described. The control group was G7. The cells were imaged at 0 h, 12 h and 24 h, and the uncovered area was calculated using Image J software.

### 4.8 Tube Formation Assay

Tube formation assays were carried out as described previously [35, 36]. HUVECs were starved for 24 h, harvested by trypsin detachment and seeded at a density of 5.10^4^ cells/well in 24‐well plates precoated with Matrigel (356234, Corning Matrigel Matrix). They were subsequently treated with G25 and/or C. G7 was a control group. The cell culture media used was supplemented with 1% FBS. The images were taken at fourfold and/or tenfold magnification using an inverted light microscope. In addition, they were taken within a time of 6–18 h and/or when the tube formation was almost complete in one of the groups to avoid possible degradation of the tubes. The extent of tube formation was quantified by counting the number of meshes using Image J software.

### 4.9 Immunofluorescence (IF)

HUVECS were seeded at a density of 5.10^4^/well in 24‐well plates with corresponding cell climbing sheets for 24 h. After serum starvation, the cells were treated with high glucose and/or cholesterol as aforementioned. The control group was G7. The immunofluorescence was therefore performed as previously described [37]. In brief, the cells were washed with PBS, fixed in 4% formaldehyde at room temperature (RT) for 15 min. They were permeabilised with 0.2% Triton X‐100 for 10 min at RT. After blocking with 5% BSA (SW3015, Solarbio)for 30 min at RT, the samples were incubated at 4°C overnight with primary antibodies against rabbit CD31 (Affinity Biosciences Cat# AF0077, RRID: https://www.antibodyregistry.org/AB_2833268), rabbit alfa‐SMA (Affinity Biosciences Cat# AF1032, RRID: https://www.antibodyregistry.org/AB_2835329) and mouse TGF Beta‐1 (Thermo Fisher Scientific Cat# MA5‐16949, RRID: https://www.antibodyregistry.org/AB_2538424). After the incubation with the primary antibodies, the cells were incubated with the fluorescent secondary antibodies. Those antibodies were Goat Anti‐Rabbit IgG H&L (Zen Bio Cat# 550043, RRID: https://www.antibodyregistry.org/AB_3668933) and Goat Anti‐Mouse IgG H&L (Zen Bio Cat# 550036, RRID: https://www.antibodyregistry.org/AB_3668934). Finally, the cells were counterstained with 4′,6‐diamidino‐2‐phenylindole (DAPI, Beyotime) and visualised on a confocal microscope (Nikon ACLIPSE Ti, Japan).

### 4.10 Statistical Analysis

At the beginning of the study, the overall estimated sample size was 882 (294 participants in each group). We used GPower 3.1 software to estimate the sample size by applying Cohen's effect size conventions [38] (Effect size = 0.12; *α* = 0.05; Power = 0.90; and Number of groups = 3). However, we finally obtained an overall sample size of 733 due to the retrospective nature of this study (some subjects have been removed by our exclusion criteria).

The continuous variables were expressed as mean ± standard deviation (mean ± SD) for data deemed to be normally distributed. They were expressed as median (interquartile range) [median (IQR)] for non‐normally distributed data. The normal distribution and equality of variance of continuous data were evaluated using the Kolmogorov–Smirnov test and Levene test, respectively. The categorical variables were expressed as percentages (%). The differences between groups were analysed by Independent *t*‐test or Analysis of Variance (one‐way ANOVA test) for data with normal distribution. They were analysed by Mann–Whitney U test or Kruskal–Wallis test when data were not normally distributed. Chi‐square test was used to analyse differences between groups for categorical variables. When a significant difference was shown between three or more groups, post hoc analyses with least significant difference test (LSD) or multiple comparison tests were also performed.

We used multiple logistic regression analysis to determine the association of circulating Nogo‐B levels with vascular complications in T2DM patients. Therefore, two main analyses were created as follows: (1) association of circulating levels of Nogo‐B with vascular complications when DM + VC group was compared with DM group; (2) association of circulating levels of Nogo‐B with vascular complications when comparing DM + VC group with NC group. Multivariate logistic regression analysis was also used to assess the association between circulating levels of Ngo‐B and T2DM for sensitivity analysis (DM group compared with NC group). Furthermore, we used logistic regression analyses to see whether the association of lower levels of Nogo‐B with vascular complications in T2DM patients was merely related to vascular disease (macro‐ or microvascular complication) when comparing DM + VC with NC. In fact, vascular complications were not matched between the two groups.

The univariate logistic regression was conducted prior to the multivariate model to select the covariates. The covariates with P value < 0.1 and/or clinically appropriate variables were considered for the multivariate model [31, 39]. Additionally, highly correlated covariates were also removed in the multivariate models. We included covariates such as traditional risk factors of T2DM and/or vasculopathy in T2DM (age, sex, BMI, hypertension, smoking, TC, HDL‐C, LDL‐C, triglycerides, smoking, alcohol intake, duration of diabetes, creatinine glycaemia and/or HbA1C), macroangiopathy and/or microangiopathy (CVD, CAD, PAD, retinopathy, DPN), cardioprotective drugs (statin and antiplatelet drugs) antihypertensive drugs (angiotensin converting enzymes inhibitors, angiotensin receptors blockers, calcium channel blockers and/or beta blockers) and oral antidiabetic drugs (metformin, DPP‐4 inhibitors and/or alfa glucosidase inhibitors). The area under the receiver operating characteristic (ROC) curves were calculated to test the discrimination of vascular complications (VC) and T2DM.

Within‐group analyses were conducted in the DM + VC group to assess the effects of individual VC on concentrations of Nog‐B. Those are complications such as DPN, retinopathy, nephropathy, history of DFU, PAD, CAD and CVD. *p* value < 0.05 was considered to be statistically significant. Graphs were created using GraphPad Prism 9.5.1 software. Image J version 1.54 g software was used to obtain image data. All the statistical analyses were performed using GraphPad or SPSS version 26 software.

## 5 Results

### 5.1 Characteristics of Subjects in the Different Groups

**FIGURE 1:**
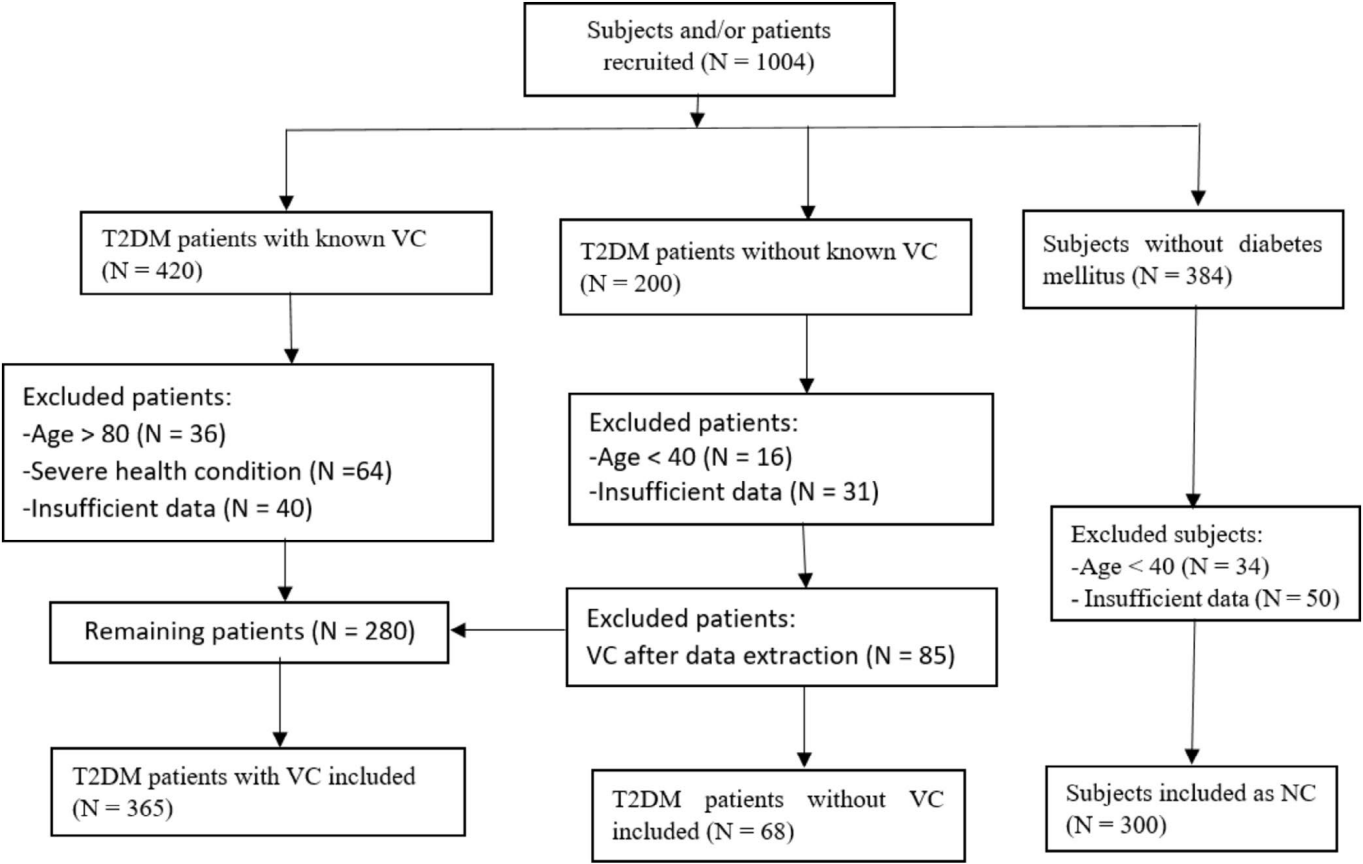
The flow chart of selection criteria.

The demographic and clinical characteristics of all subjects are summarised in Table 1. The flow chart of selection criteria is shown in Figure 1. In DM + VC group, all the participants had at least one macroangiopathy and/or one microangiopathy such as DPN, diabetic nephropathy (DN) or CKD, retinopathy, PAD and/or DFU, CVD and CAD. Smoking and sex rates were similar in DM and NC groups. The means for age and BMI were also similar in DM and NC groups. However, patients in DM + VC group were significantly older and showed a significantly higher proportion of males when compared with those in DM or NC groups. DM or NC group displayed significantly higher rates of smoking than DM + VC group. DM + VC showed a significantly longer duration of diabetes when compared with DM. DM + VC also displayed higher rates of medication including statins, antiplatelet and antihypertensive drugs.

**TABLE 1:** Demographic and clinical data of participants in different groups.

Variables	NC (*N* = 300)	DM (*N* = 68)	DM + VC (*N* = 365)	*p*
Male, *n* (%)	169 (56.3)	38 (55.9)^c^	262 (71.8)	< 0.001
Age (Years)	54.08 ± 9.95	56.01 ± 8.27^c^	62.64 ± 10.04	< 0.001
BMI (kg/m^2^)	24.22 ± 2.94	23.32 ± 3.01^c^	23.22 ± 3.27^b^	0.003
Smoking, current/past, *n* (%)	225 (75)	50 (73.5)^c^	180 (49.5)	< 0.001
Heavy drinking, current/past, *n* (%)	124 (41.3)	30 (44.1)	139 (38.2)	0.54
Duration of T2DM (years)	—	0.6 (0.2–5)	10 (4–16)	< 0.001
Metformin, *n* (%)	—	66 (97.1)	194 (53.1)	< 0.001
DPP‐4 inhibitors, *n* (%)	—	—	107 (29.3)	N/A
α‐glucosidase inhibitors, *n* (%)	—	2 (2.9)	45 (12.3)	0.02
SGLT‐2 inhibitors, *n* (%)	—	—	218 (59.7)	N/A
GLP‐1 agonists, *n* (%)	—	9 (13.2)	26 (7.1)	0.189
Oral hypoglycemic agents, *n* (%)	—	66 (97.1)	309 (84.6)	0.006
Insulin, *n* (%)	—	—	265 (72.6)	N/A
Statins, *n* (%)	91 (30.4)	—	300 (82.2)	< 0.001
Antiplatelet drugs, *n* (%)	82 (27.3)	—	216 (59.2)	< 0.001
Antihypertensive drugs, *n* (%)	67 (22.3)	13 (19.1)^c^	131 (36)	< 0.001
Hypertension, *n* (%)	73 (24.3)	15 (22.1)^c^	187 (51.2)	< 0.001
Hypertension with retinopathy, *n* (%)	16 (5.4)	—	—	N/A
Fasting Glycaemia (mmol/L)	5.3 (5.1–5.6)	8.6 (7.1–10.95)	7.7 (6.6–9.6)	< 0.001
HbA1C (%)	5.5 (5–5.7)	7.5 (6.8–9.6)	8.5 (7.3–10.6)^b^	< 0.001
ALT (U/L)	20 (14–26)	23.5 (17–33.5)	16 (11–25)	< 0.001
AST (U/L)	21 (19–25)	22.5 (17–27)^c^	18 (14–25.25)	< 0.001
Total Cholesterol (mmol/L)	5.22 (4.5–5.75)	4.73 (4.13–5.36)	3.76 (3.17–4.52)	< 0.001
Triglycerides (mmol/L)	1.34 (0.86–1.92)	2.36 (1.57–3.2)	1.3 (0.97–1.95)^a^	< 0.001
HDL‐Cholesterol (mmol/L)	1.4 (1.17–1.69)	1.09 (1–1.35)	0.96 (0.79–1.16)	< 0.001
LDL‐Cholesterol (mmol/L)	2.96 (2.47–3.51)	2.59 (1.4–3.15)	2.22 (1.61–2.81)^b^	< 0.001
Uric Acid (μmol/L)	317.22 ± 81.10	302.21 ± 75.44	316.36 ± 99.18	0.59
Creatinine (μmol/L)	73.9 ± 17.68	68.72 ± 19.97^c^	86.84 ± 41.93	< 0.001
Albuminuria, *n* (%)	—	—	201 (56)	N/A
ABI	1.07 (1.03–1.13)	1.08 (1–1.2)	1.11 (0.94–1.19)	0.11
Nephropathy/CKD, *n* (%)	—	—	219 (60)	N/A
Retinopathy, *n* (%)	16 (5.4)	—	120 (32.9)	< 0.001
DPN (distal peripheral neuropathy), *n* (%)	27 (9)	—	306 (84.3)	< 0.001
History of DFU, *n* (%)	—	—	250 (68.5)	N/A
LEAD (PAD), *n* (%)	113 (38)	—	291 (79.7)	< 0.001
CVD, *n* (%)	127 (42.6)	—	258 (70.7)	< 0.001
CAD, *n* (%)	121 (40.5)	—	258 (70.7)	< 0.001
Nogo‐B levels (ng/mL)	34.4 ± 5.03	31.04 ± 4.28	24.19 ± 5.24	< 0.001

Abbreviation: N/A, non applicable.

^a^ *p* > 0.05 compared with NC.

^b^ *p* > 0.05 compared with DM.

^c^ *p* > 0.05 compared with NC.

In all the groups assessed, ABI was similar. HbA1C was similar in DM + VC and DM groups. The rates of antihypertensive drugs and hypertension were similar in DM and NC. However, DM + VC displayed significantly lower levels of TC and HDL‐C than NC or DM. Of the T2DM patients with VC, 68.5% had a history of DFU.

**FIGURE 2:**
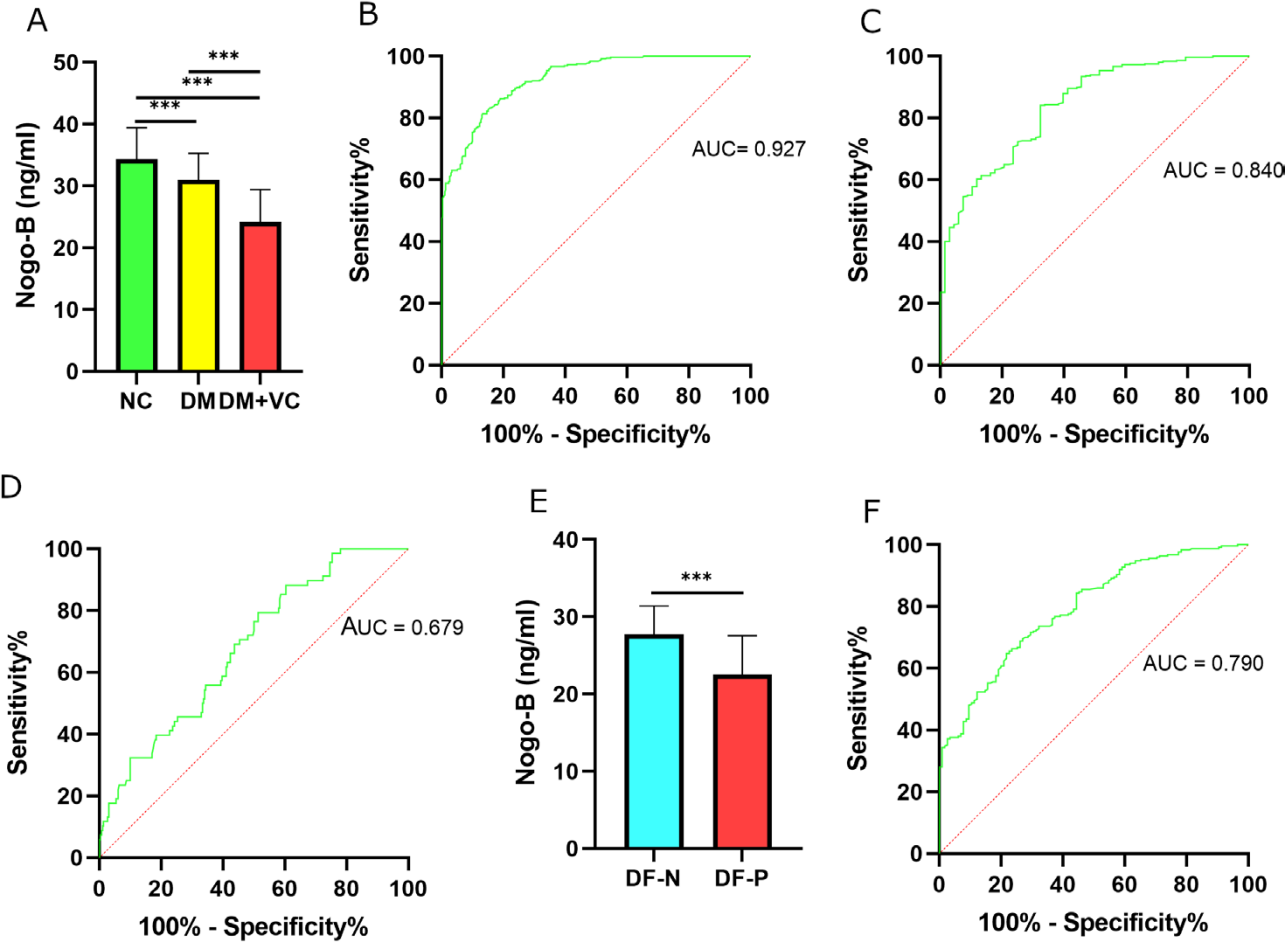
Serum Nogo‐B levels and ROC curves. (A) Comparison of circulating Nogo‐B levels across the groups (NC, DM and DM + VC). (B) Lower levels of circulating Nogo‐B have the capability for the determination of a vascular disease in T2DM patients when comparing DM + VC with NC (AUC = 0.927). (C) Lower levels of Nogo‐B displayed the discriminative capability for the determination of VC when DM + VC was compared with DM (AUC = 0.840). (D) Evaluation of the discriminative capability of lower levels of Nogo‐B to distinguish DM from NC (AUC = 0.679). (E) Comparison of Nogo‐B levels between DF‐P and DF‐N. (F) Lower levels of Nogo‐B exhibited the capability for the determination of history of DFU when comparing DF‐P with DF‐N (AUC: 0.790). Data are expressed as mean ± standard deviation (*M* ± SD). ***p < 0.001.

Patients in DM + VC displayed significantly lower levels of circulating Nogo‐B when compared with those in DM or NC (*p* < 0.001). In addition, circulating levels of Nogo‐B were significantly lower in DM than NC (*p* < 0.001). Levels of Nogo‐B in all the groups are illustrated in Figure 2A.

### 5.2 Association of Levels of Circulating Nogo‐B With VC

#### 5.2.1 Correlation of Lower Levels of Circulating Nogo‐B With VC When Comparing DM + VC With NC

The association of lower levels of circulating Nogo‐B with VC was analysed using multiple logistic regression (Table 2). Low levels of circulating Nogo‐B were strongly associated with VC in crude model [Odds ratio (OR) = 0.630, *p* < 0.001]. This association remained statistically significant even after adjustment for age, sex, hypertension, BMI, smoking, TC, HDL‐C, LDL‐C and creatinine (Model 3, OR = 0.649, *p* < 0.001). Furthermore, ROC curve was created to evaluate the discriminative capability of circulating levels of Nogo‐B for the determination of VC in T2DM (Figure 2B). The findings indicated that a cut‐off value of 28.82 ng/mL had high sensitivity (83.56%) and specificity (83.67%) to distinguish VC from non‐VC. The Area under curve (AUC) [95% confidence interval (CI)] was 0.927 (0.909–0.945), with *p* < 0.001.

**TABLE 2:** Association of lower levels of circulating Nogo‐B with VC when DM + VC was compared with NC.

Models	Independent variable	OR (95% CI)	*p*
Model 1	Nogo‐B (ng/mL)	0.630 (0.587–0.678)	< 0.001
Model 2	Nogo‐B (ng/mL)	0.637 (0.590–0.687)	< 0.001
Model 3	Nogo‐B (ng/mL)	0.649 (0.579–0.729)	< 0.001

Abbreviations: Model 1: Crude; Model 2: adjusted for age, sex, hypertension; Model 3: Adjusted for, age, sex, hypertension, BMI, smoking, TC, HDL‐C, LDL‐C and creatinine.

#### 5.2.2 Association of Lower Levels of Nogo‐B With VC When DM + VC Was Compared With DM

Multiple logistic regression was conducted to determine the association between low levels of Nogo‐B and VC (Table 3). Lower levels of Nogo‐B were significantly associated with VC in the crude model (OR = 0.719, *p* < 0.001). The association was still statistically significant after adjustment for age, sex, duration of diabetes, hypertension, smoking, TC, triglyceride, HDL‐C, creatinine, glycemia and HbA1C (Model 3, OR = 0.705, *p* < 0.001). According to ROC curve analysis, the AUC (95% CI) of circulating levels of Nogo‐B in differentiating DM + VC from DM was 0.840 (0.791–0.889) (Figure 2C). A cut‐off value of 27.68 ng/mL had sensitivity and specificity of 72.6% and 73.53%, respectively.

**TABLE 3:** Association of lower levels of circulating Nogo‐B with VC when DM + VC was compared with DM.

Models	Independent variable	OR (95% CI)	*p*
Model 1	Nogo‐B (ng/mL)	0.719 (0.664–0.780)	< 0.001
Model 2	Nogo‐B (ng/mL)	0.727 (0.662–0.799)	< 0.001
Model 3	Nogo‐B (ng/mL)	0.705 (0.596–0.834)	< 0.001

Abbreviations: Model 1: Crude; Model 2: adjusted for age, sex and duration of diabetes; Model 3: Adjusted for age, sex, duration of diabetes, hypertension, smoking, TC, triglyceride, HDL‐C, creatinine, glycaemia and HbA1C.

#### 5.2.3 Association of Lower Levels of Serum Nogo‐B With Diabetes When DM Group Was Compared With NC

Finally, a multiple logistic regression was performed to analyse the association between lower levels of circulating Nogo‐B and T2DM for sensitivity analysis (Table 4). Lower levels of circulating Nogo‐B were significantly associated with T2DM (OR = 0.861, *p* < 0.001). This association remained statistically significant even after adjustment for BMI, TC, triglyceride, HDL‐C, LDL‐C and creatinine (Model 3, OR = 0.826, *p* = 0.026). ROC curve analysis was also used to evaluate the discriminative capability of circulating levels of Nogo‐B in order to distinguish DM from NC (Figure 2D). The findings showed that the sensitivity and specificity for a cut‐off value of 33.1 ng/mL were 61.76% and 59%, respectively. The AUC (95% CI) was 0.679 (0.613–0.745), with *p* < 0.001.

**TABLE 4:** Association of lower levels of Nogo‐B with DM when comparing DM with NC.

Models	Independent variable	OR (95% CI)	*p*
Model 1	Nogo‐B (ng/mL)	0.861 (0.810–0.916)	< 0.001
Model 2	Nogo‐B (ng/mL)	0.835 (0.728–0.957)	0.01
Model 3	Nogo‐B (ng/mL)	0.826 (0.698–0.977)	0.026

Abbreviations: Model 1: Crude; Model 2: adjusted for BMI, TC and triglycerides; Model 3: adjusted for BMI, TC, triglyceride, HDL‐C, LDL‐C and creatinine.

#### 5.2.4 Lower Levels of Nogo‐B Are Not Merely Related to Macrovascular Disease in T2DM Patients When Comparing DM + VC With NC

By univariate analyses, macrovascular disease (PAD, CVD and CAD) strongly correlated with VC in T2DM patients when comparing DM + VC with NC. To assess whether lower levels of Nogo‐B were not exclusively associated with macrovascular complications in DM + VC, Nogo‐B levels were adjusted for macrovascular complications by logistic regression analyses (Table 5). The results showed that lower levels of Nogo‐B remained significantly associated with VC in T2DM (OR: 0.636; 95% CI: 0.588, 0.687; *p* < 0.001).

**TABLE 5:** Lower levels of Nogo‐B still correlated with VC in T2DM patients after adjustment for macroangiopathy.

Model	Independent variable	OR (95% CI)	*p*
Univariate analysis	CVD	3.247 (2.353–4.479)	< 0.001
Univariate analysis	CAD	3.547 (2.569–4.898)	< 0.001
Univariate analysis	PAD	6.403 (4.529–9.054)	< 0.001
Multivariate analysis	Nogo‐B (ng/mL)^a^	0.636 (0.588–0.687)	< 0.001

^a^ Adjusted for CVD, CAD and PAD.

#### 5.2.5 Lower Levels of Nogo‐B Were Not Exclusively Related to DPN and/or Retinopathy in T2DM Patients When Comparing DM + VC With NC

Retinopathy and DPN were associated with VC by univariate analyses. We sought to know whether lower levels of Nogo‐B were still associated with VC after adjustment for retinopathy and DPN (Table 6). The results showed that lower levels of Nogo‐B were still significantly associated with VC in T2DM when comparing DM + VC with NC (OR: 0.646; 95% CI: 0.588, 0.710; *p* < 0.001).

**TABLE 6:** Lower levels of Nogo‐B are not merely related to Retinopathy and/or DPN in T2DM patients.

Model	Independent variable	OR (95% CI)	*p*
Univariate analysis	Retinopathy	8.663 (5.003–15)	< 0.001
Univariate analysis	DPN	54.281 (33.384–88.259)	< 0.001
Multivariate analysis	Nogo‐B (ng/mL)^a^	0.646 (0.588–0.710)	< 0.001

^a^ Adjusted for retinopathy and DPN.

#### 5.2.6 Lower Levels of Nogo‐B Are Still Associated With VC in T2DM After Adjustments of Vascular Protective Drugs When Comparing DM + VC With NC

Treatments such as OAD and insulin were not significantly associated with VC in T2DM, by univariate analyses. However, statin, antiplatelet and antihypertensive drugs were associated with VC in T2DM patients (Table 7). To assess whether these vascular protective drugs affected the association of lower levels of Nogo‐B and VC in T2DM, logistic regression was performed. Lower levels of Nogo‐B remained significantly associated with VC in T2DM after adjustment of vascular protective drugs (OR: 0.635; 95% CI: 0.587, 0.688; *p* < 0.001). This means that vascular protective drugs might not significantly change the association of lower levels of Nogo‐B with vascular diseases in T2DM.

**TABLE 7:** Cardioprotective drugs did not affect the association of lower levels of Nogo‐B with VC in diabetes.

Model	Independent variable	OR (95% CI)	*p*
Univariate analysis	Statin treatment	10.715 (7.435–15.440)	< 0.001
Univariate analysis	Antiplatelet treatment	3.919 (2.824–5.44)	< 0.001
Univariate analysis	Antihypertensive drugs	1.955 (1.384–2.763)	< 0.001
Multivariate analysis	Nogo‐B (ng/mL)^a^	0.635 (0.587–0.688)	< 0.001

^a^ Adjusted for statin treatment, antiplatelet treatment and antihypertensive drugs.

#### 5.2.7 Lower Levels of Nogo‐B Are Still Associated With VC in T2DM Patients After Adjustment for HbA1C, When DM + VC Was Compared With DM

HbA1C was positively associated with VC by univariate analyses. We consequently analysed whether adjustment for HbA1C affected the association of lower levels of Nogo‐B with VC (Table 8). This association remained statistically significant even after adjustment for HbA1C (OR: 0.72; 95% CI: 0.66–0.78).

**TABLE 8:** Lower levels of Nogo‐B are still associated with VC in diabetes after adjustment for HbA1C.

Model	Independent variable	OR (95% CI)	*p*
Univariate analysis	HbA1C	1.18 (1.03–1.36)	0.02
Multivariate analysis	Nogo‐B (ng/mL)^a^	0.72 (0.66–0.78)	< 0.001

^a^ Adjusted for HbA1C.

#### 5.2.8 Lower Levels of Nogo‐B Are Still Associated With VC After Adjustment for Antihypertensive Drugs When DM + VC Is Compared With DM

Statin and antiplatelet drugs were not significantly associated with VC by univariate analyses. However, antihypertensive drugs were associated with VC in T2DM patients (Table 9). We therefore assessed whether antihypertensive drugs affected the association of lower levels of Nogo‐B with VC in T2DM patients. After their adjustment, lower levels of Nogo‐B remained associated with VC in T2DM (OR: 0.706; 95% CI: 0.648, 0.768; *p* < 0.001).

**TABLE 9:** Antihypertensive drugs did not change the association of lower levels of Nogo‐B with vascular disease.

Model	Independent variable	OR (95% CI)	*p*
Univariate analysis	Antihypertensive drugs	2.379 (1.253–4.517)	0.008
Multivariate analysis	Nogo‐B (ng/mL)^a^	0.706 (0.648–0.768)	< 0.001

^a^ Adjusted for antihypertensive drugs.

#### 5.2.9 Lower Levels of Nogo‐B Still Correlated With VC in T2DM After Adjustment for OAD When Comparing DM + VC With DM

OAD were significantly associated with VC by univariate analyses (Table 10). We assessed whether OAD affected the correlation of lower levels of Nogo‐B with VC in T2DM. By multivariate analysis, lower levels of Nogo‐B were still associated with VC in T2DM patients after adjustment for OAD (OR: 0.716, 95% CI: 0.659, 0.778, *p* < 0.001).

**TABLE 10:** Lower levels of Nogo‐B were still associated with VC after adjustment for OAD.

Model	Independent variable	OR (95% CI)	*p*
Univariate	Metformin	28.92 (6.98–119.84)	0.001
Univariate	Alfa‐glucosidase inhibitors	4.65 (1.1–19.6)	0.036
Univariate analysis	OAD (combined)	0.17 (0.041–0.715)	0.016
Multivariate analysis	Nogo‐B (ng/mL)^a^	0.723 (0.667–0.784)	< 0.001

^a^ Adjusted for OAD.

#### 5.2.10 Lower Levels of Nogo‐B Are Not Merely Related to Glycaemia in T2DM Patients Without VC

Glycaemia was strongly correlated to T2DM (DM) by univariate analyses (Table 11). We intended to know whether the association of lower levels of Nogo‐B with T2DM was merely exclusively to glycaemia. Lower levels of Nogo‐B still correlated with T2DM after adjustment of glycaemia (OR: 0.873; 95% CI: 0.766, 0.966; *p* = 0.043). These findings mean that factors other than diabetes play a role in the reduction of Nogo‐B in T2DM patients without VC.

**TABLE 11:** Lower levels of Nogo‐B are not only related to glycaemia in T2DM patients without VC.

Model	Independent variable	OR (95% CI)	*p*
Univariate analysis	Glycaemia (mmol/L)	12.549 (6.286–25.050)	< 0.001
Multivariate analysis	Nogo‐B (ng/mL)^a^	0.873 (0.766–0.996)	0.043

^a^ Adjusted for glycaemia.

### 5.3 Within Group Analyses

In DM + VC, patients with history of DFU (DF‐P) displayed significantly lower levels of circulating Nogo‐B than those without DFU (DF‐N) (Figure 2E and Table 12). This difference remained statistically significant even after adjustment for factors known to be associated with DFU (Table 13). ROC curve analysis was also used to evaluate the discriminative capability of circulating levels of Nogo‐B in order to distinguish DF‐P from DF‐N (Figure 2F). The findings showed that the sensitivity and specificity for a cut‐off value of 25.63 ng/mL were 70% and 71.34%, respectively. The AUC (95% CI) was 0.790 (0.747–0.840), with *p* < 0.001. Patients with history of PAD exhibited lower levels of Nogo‐B than those without PAD (*p* = 0.02). However, this difference did not remain significant after adjustment for history of DFU (*p* = 0.33). Therefore, in DM + VC, the association of lower levels of Nogo‐B with PAD was merely related to history of DFU. No significant differences in Nogo‐B levels were shown between patients with a specific complication and patients without it in other types of complications (Table 12).

**TABLE 12:** The comparison of effects of individual vascular complications on Nogo‐B concentration.

Name of the complication	Nogo‐B levels in patients with the complication (ng/mL)	Nogo‐B levels in patients without the complication (ng/mL)	*p*
DN/CKD	24.2 ± 5.15	24.18 ± 5.4	0.958
Retinopathy	23.43 ± 6.02	24.57 ± 4.8	0.05
DPN	23.97 ± 5.21	25.3 ± 5.41	0.086
PAD	23.87 ± 5.19	25.47 ± 5.31	0.019
DFU	22.54 ± 5.05	27.79 ± 3.63	**< 0.001**
CAD	24.08 ± 5.24	24.46 ± 5.29	0.53
CVD	24.44 ± 5.25	23.6 ± 5.22	0.166

*Note:* Bold values indicate PAD or DFU showed a further low levels of Nogo‐B than other complications.

**TABLE 13:** The association of lower levels of Nogo‐B with history of DFU in T2DM patients.

Model	Independent variable	OR (95% CI)	*p*
Model 1	Nogo‐B (ng/mL)	0.751 (0.699–0.807)	< 0.001
Model 2	Nogo‐B (ng/mL)	0.756 (0.703–814)	< 0.001
Model 3	Nogo‐B (ng/mL)	0.760 (0.703–0.823)	< 0.001

Abbreviations: Model 1: crude model; Model 2: adjusted for DPN, PAD and HDL‐C; Model 3: adjusted for DPN, PAD, HDL‐C, smoking, alcohol intake, TC, Triglyceride, retinopathy and sex.

### 5.4 IHC

**FIGURE 3:**
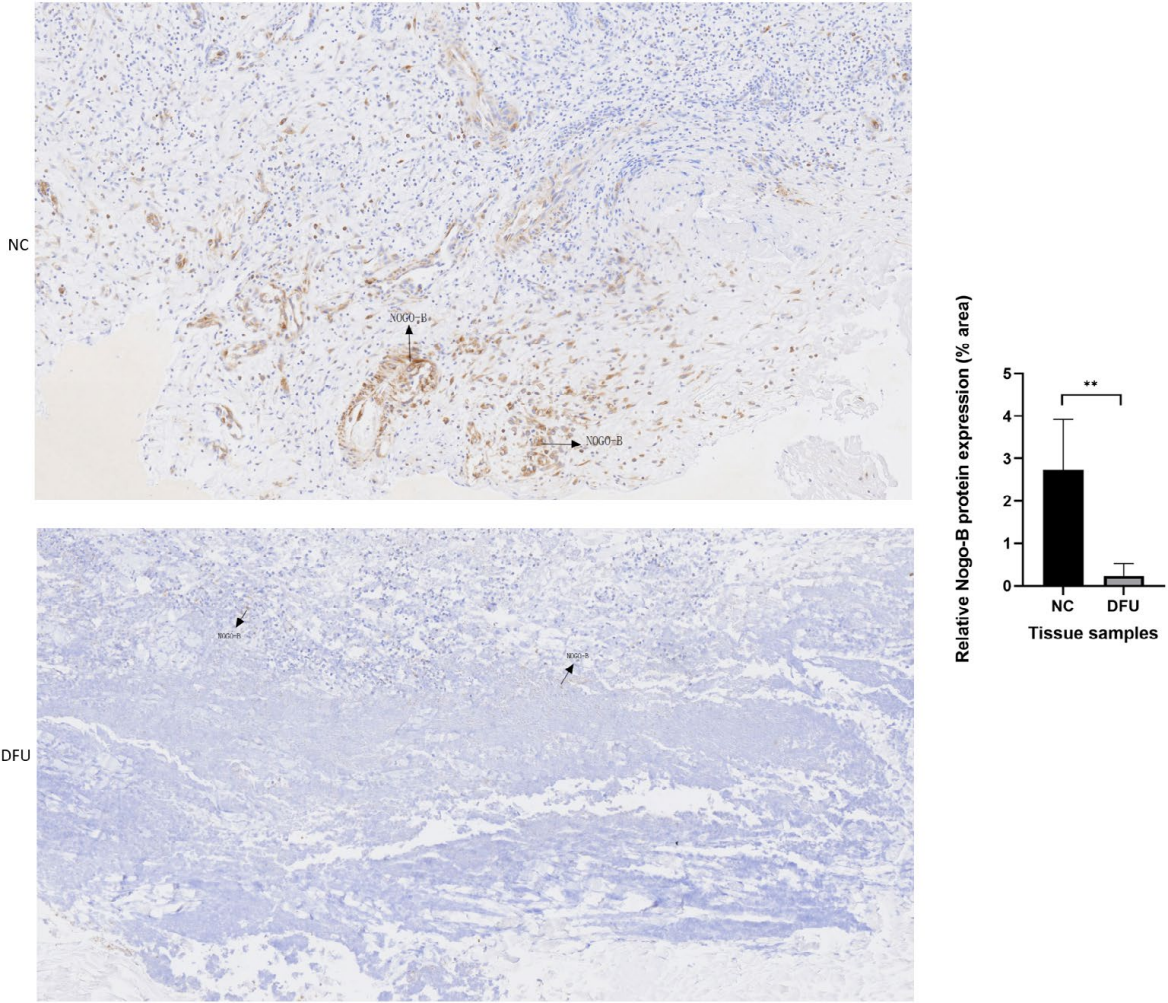
Immunohistochemistry analysis showing the expression of Nogo‐B in subcutaneous tissues from feet of patients with or without DFU. The control group is shown as NC. Data are expressed as *M* ± SD. Arrows represent Nogo‐B expression. **p < 0.01, compared with NC.

The results of IHC analyses showed that the expression of Nogo‐B significantly decreased in subcutaneous tissues taken from the feet of patients with DFU (*p* < 0.01). The illustrative figure is shown in Figure 3.

### 5.5 High Glucose and/or Cholesterol Induced Injury in HUVECS

**FIGURE 4:**
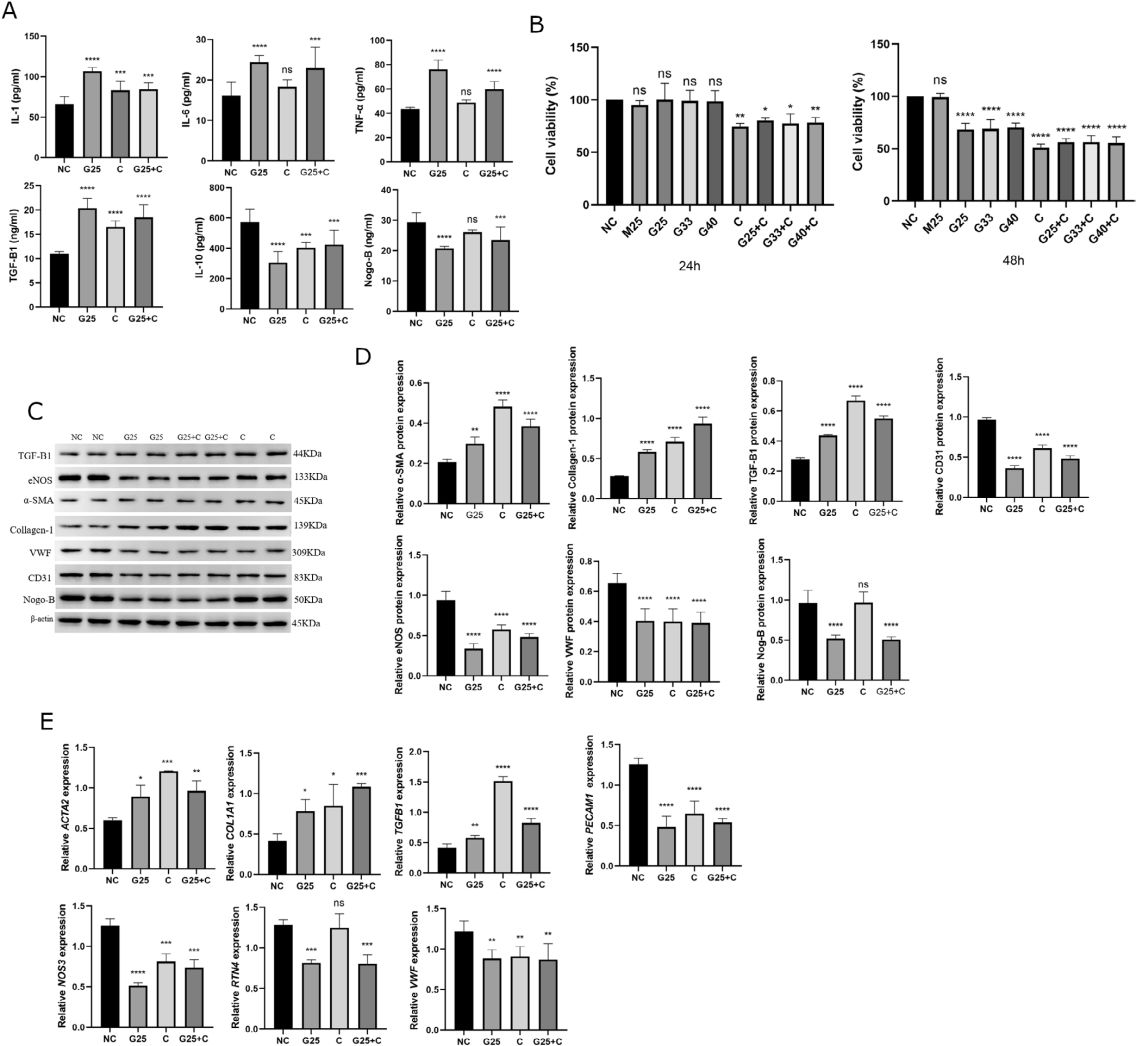
High glucose induced injury and reduction of Nogo‐B levels in HUVECs. (A) The cell culture supernatant of HUVECs treated with HG and/or C displayed an increase in inflammatory cytokines (IL‐1, IL‐6 and TNF‐α) and TGF‐β1, while there was a simultaneous decrease in IL‐10 and Nogo‐B. (B) HUVECs treated with HG and/or C exhibited a decrease of cell viability after 24 and/or 48 h. (C, D) HUVECs treated with HG and/or C increased the expression of mesenchymal marker proteins (α‐SMA and collagen‐1) and TGF‐β1, while the expression of endothelial marker proteins (CD31, eNOS and VWF) and Nogo‐B decreased. Protein levels were normalised to those of β‐Actin. (E) HUVECs treated with HG and/or C increased the expression of mesenchymal marker genes (ACTA2 and COL1A1) and TGFB1, while there was a simultaneous decrease in the expression of endothelial marker genes (PECAM1, NOS3 and VWF) and RTN4. The mRNA expression was normalised to *ACTB* (β‐Actin). Data are expressed as *M* ± SD. ****p < 0001, ***p < 0.001, **p < 0.01. *p < 0.05, ^ns^ *p* > 0.05. Data are expressed as *M* ± SD. ****p < 0.0001, **p < 0.01, *p < 0.05, ^ns^ *p* > 0.05.

To mimic the vascular endothelial cell injury in diabetes, HUVECs were treated with high glucose (HG) and/or cholesterol (C). The cell culture supernatant displayed a significant increase in inflammatory cytokines (IL‐1, IL‐6 and TNF‐α) and TGF‐β1, while displaying a decrease in the levels of IL‐10 (Figure 4A). In addition, HG and/or C in HUVECs induced a significant decrease in cell viability (Figure 4B).

### 5.6 Nogo‐B Levels Decreased in HUVECs Treated With HG and/or C

The cell culture supernatant of HUVECs treated with HG and/or C showed a significant decrease in levels of Nogo‐B (Figure 4A). In addition, there was a significant reduction of Nogo‐B (*RTN4*) expression (Figure 4C–E).

### 5.7 HG And/or C Induced EndMT in HUVECs Treated With HG and/or C

The treatment of HUVECs with HG and/or C induced an increase in the expression and/or amount of mesenchymal marker proteins (alfa‐SMA and collagen‐1) and TGF‐B1 (Figure 4C,D). There was also a simultaneous decrease in the expression of endothelial marker proteins (CD31, eNOS and VWF) (Figure 4C,D). The same trends were found for mesenchymal and endothelial marker genes and *TGFB1* (Figure 4E).

### 5.8 Nogo‐B Knockdown Exacerbated Injury While Further Reducing the Levels or Expression of Nogo‐B, in HUVEC Treated With HG and/or C

**FIGURE 5:**
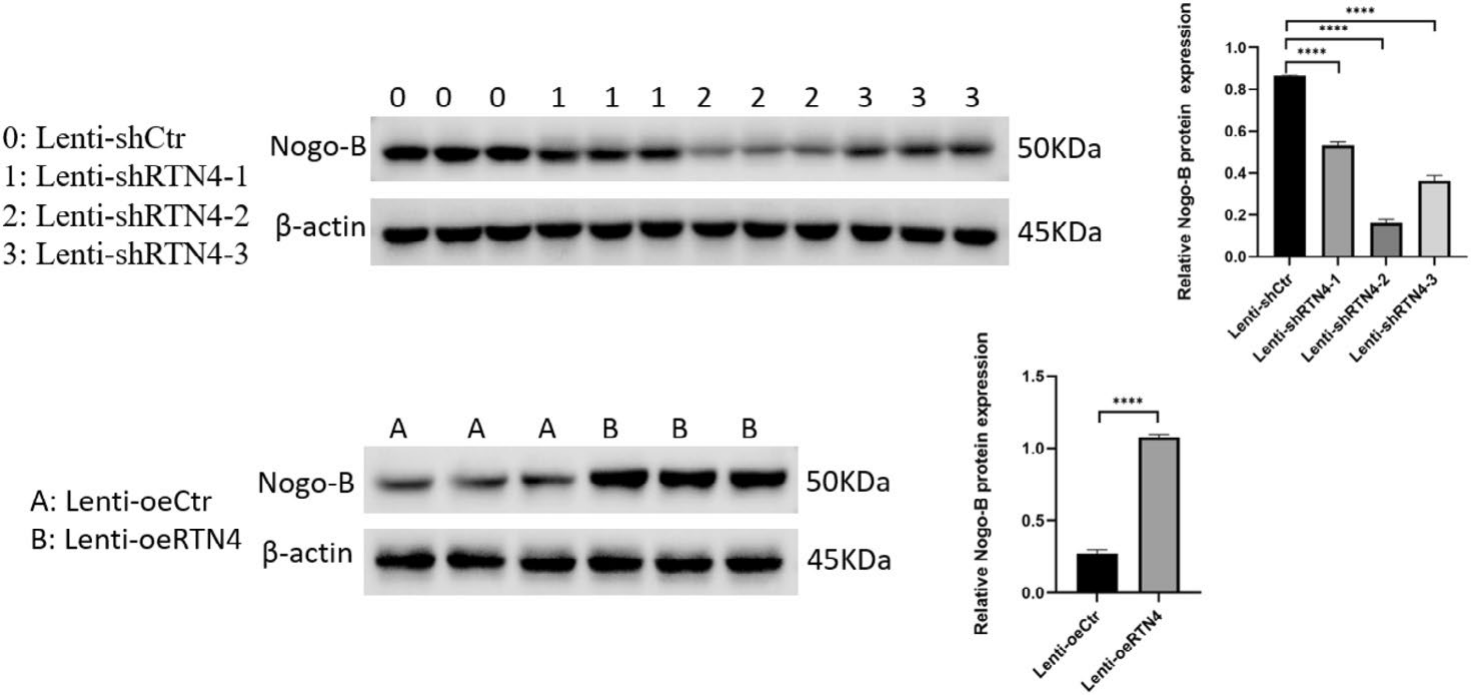
The testing of Nogo‐B knockdown and/or overexpression by WB. Data are expressed as *M* ± SD. ****p < 0.0001, expression of Nogo‐B compared with vehicle lentivirus.

**FIGURE 6:**
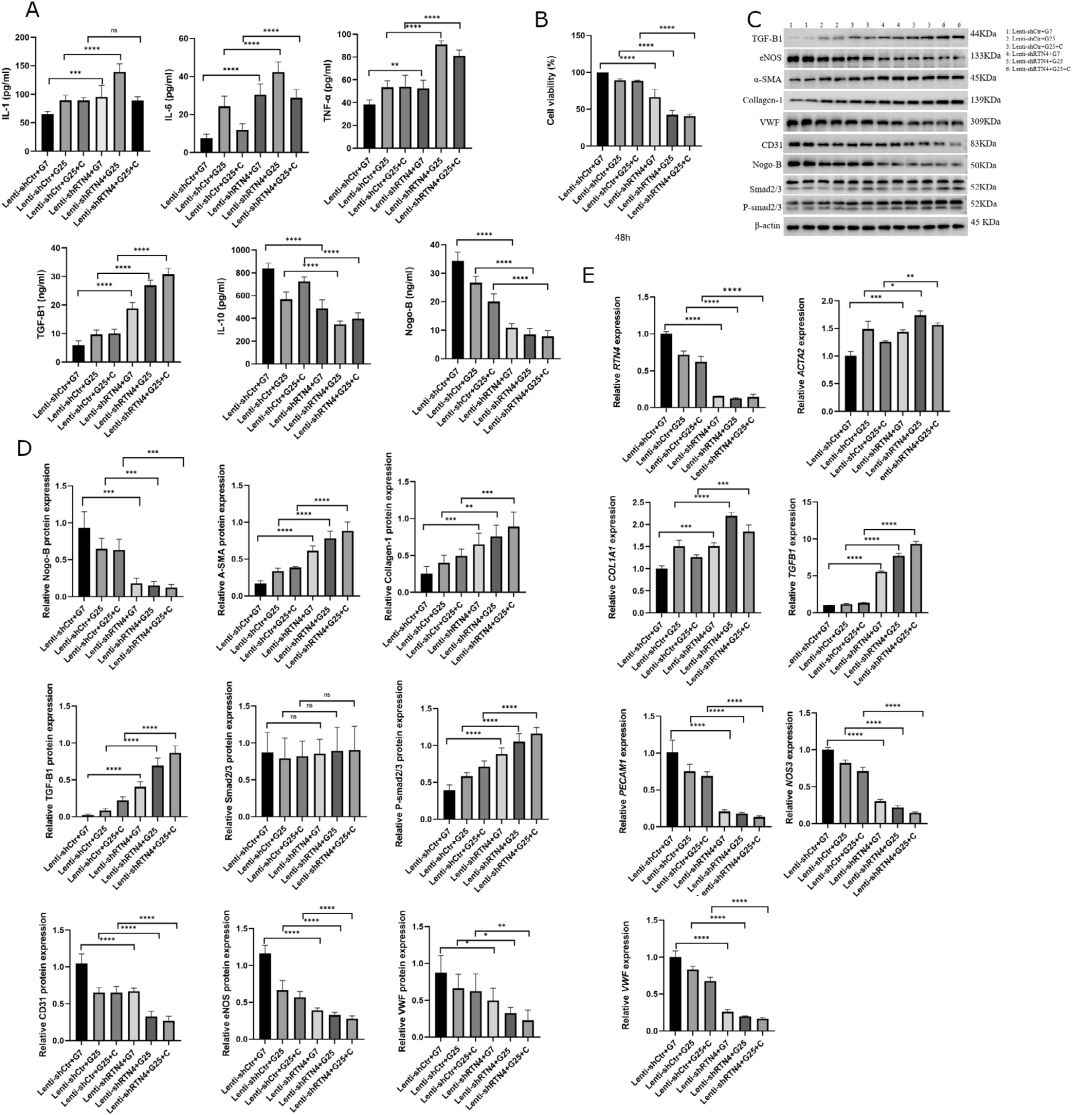
Nogo‐B knockdown exacerbated endothelial cell injury. (A) Nogo‐B knockdown further increased the levels of inflammatory cytokines and TGF‐β1 while further reducing the levels of IL‐10 and Nogo‐B, in the cell culture supernatant of HUVECs treated with HG and/or C. (B) Nogo‐B knockdown further reduced the cell viability of HUVECs under hyperglycemic condition. (C, D) Nogo‐B knockdown further increased the expression of mesenchymal marker proteins, TGF‐β1 and P‐smad2/3 while further reducing the expression of endothelial marker proteins and Nogo‐B, in HUVECs treated with HG and/or C. (E) RTN4 knockdown further increased the expression of mesenchymal marker genes and TGFB1 while it further reduced the expression of endothelial marker genes, in HUVECs treated with HG and/C. Data are expressed as *M* ± SD. ****p < 0.0001, **p < 0.01, *p < 0.05, ^ns^ *p* > 0.05.

**FIGURE 7:**
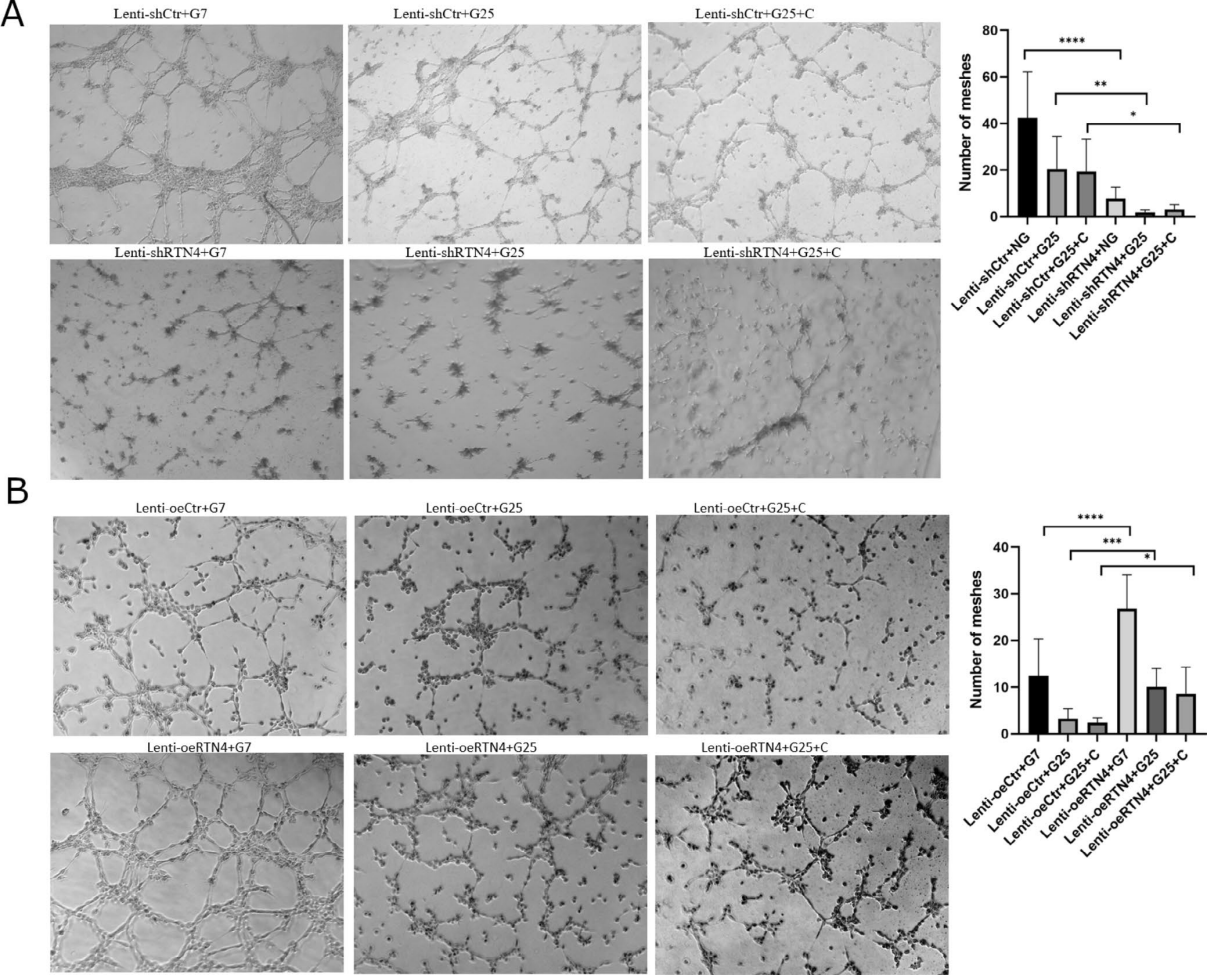
Tube formation assay. (A) Nogo‐B knockdown exhibited a further decrease of meshes in HUVECs under hyperglycemic state. (B) Nogo‐B overexpression improved the formation of tubes, in HUVECs treated with HG and/or C. Data are expressed as *M* ± SD. ****p < 0.0001, ***p < 0.001, **p < 0.01, *p < 0.05.

**FIGURE 8:**
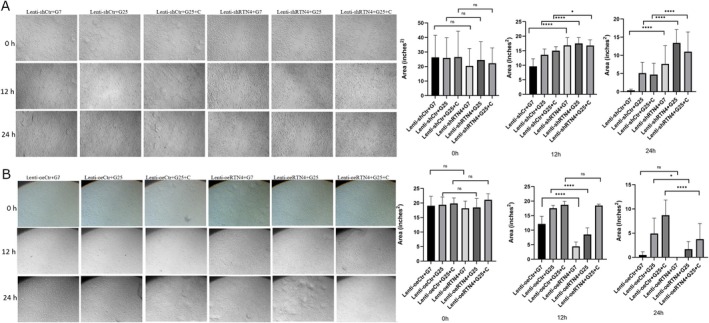
Wound healing Assay. (A) Nogo‐B knockdown further delayed wound healing in HUVECs treated with HG and/or C. (B) Nogo‐B overexpression reduced the wound healing time in HUVECs under hyperglycemic condition. Data are expressed as *M* ± SD. ****p < 0.0001, *p < 0.05, ^ns^ *p* > 0.05.

**FIGURE 9:**
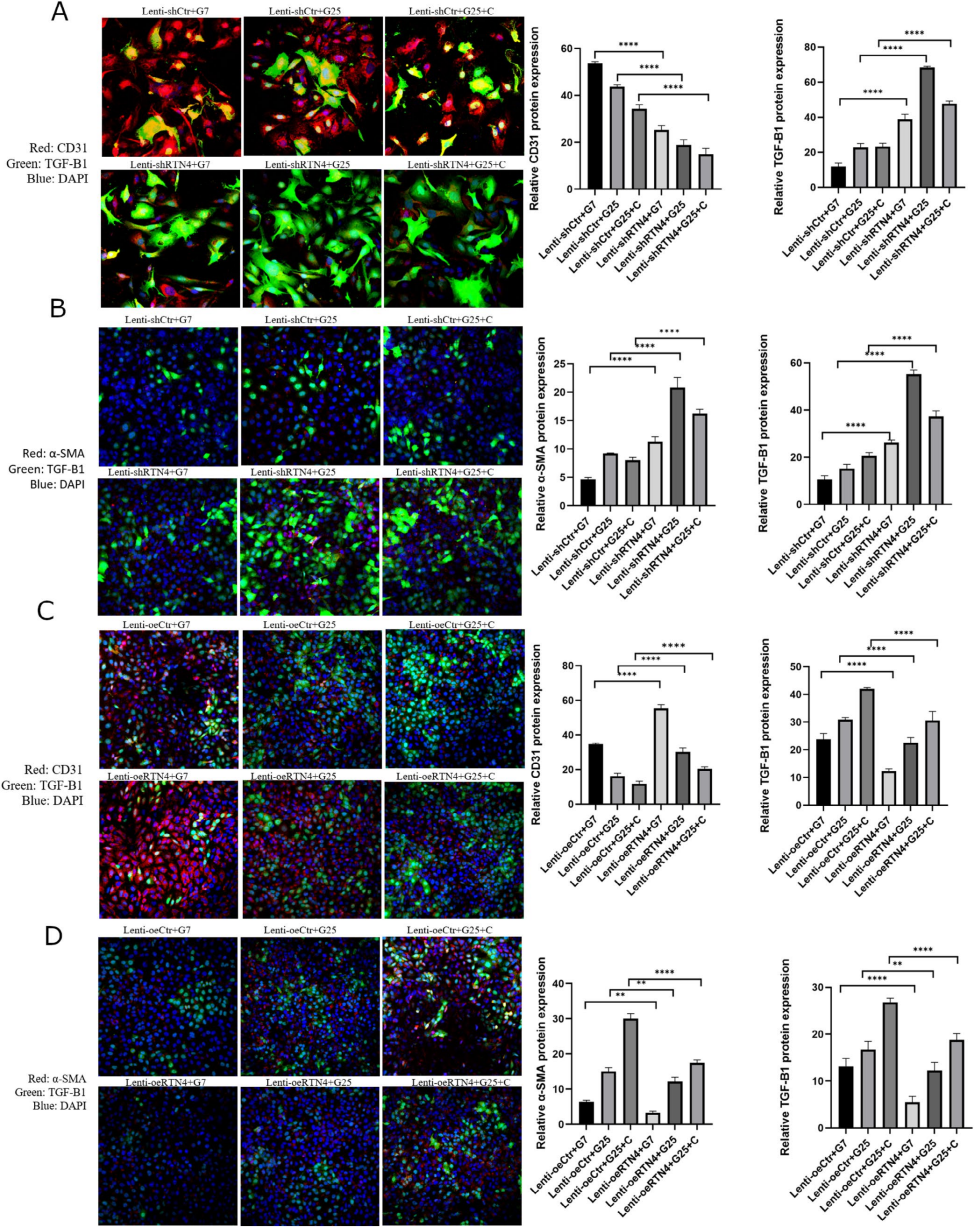
IF analyses after Nogo‐B knockdown and/or overexpression in HUVECs. (A, B) Nogo‐B knockdown further reduced the expression of CD31 while further increasing the expression of TGF‐β1 and α‐SMA, in HUVECs under hyperglycemic state. (C, D) Nogo‐B overexpression increased the expression of CD31 while there was a decrease of TGF‐β1 and α‐SMA, in HUVECs treated with HG and/or C. Data are expressed as *M* ± SD. ****p < 0.0001, **p < 0.01.

Nogo‐B silencing was tested by WB (Figure 5). Nogo‐B knockdown further increased the levels of inflammatory cytokines and TGF‐B1 in the cell culture supernatant of HUVECs treated with HG and/or C (Figure 6A). At the same time, there was a further decrease in levels of IL‐10 and Nogo‐B (Figure 6A). This was followed by a further decrease in cell viability and tube formation (Figures 6B and 7A, respectively). Nogo‐B knockdown also further delayed wound healing (Figure 8A). On the other hand, Nogo‐B knockdown worsened EndMT in HUVECs treated with HG and/or C (Figures 6C–E and 9A,B). In fact, this was characterised by a further increase in mesenchymal markers and decrease in endothelial markers (Figure 9A,B).

### 5.9 Nogo‐B Overexpression Alleviated Injury in HUVECs Treated With HG and/or C

**FIGURE 10:**
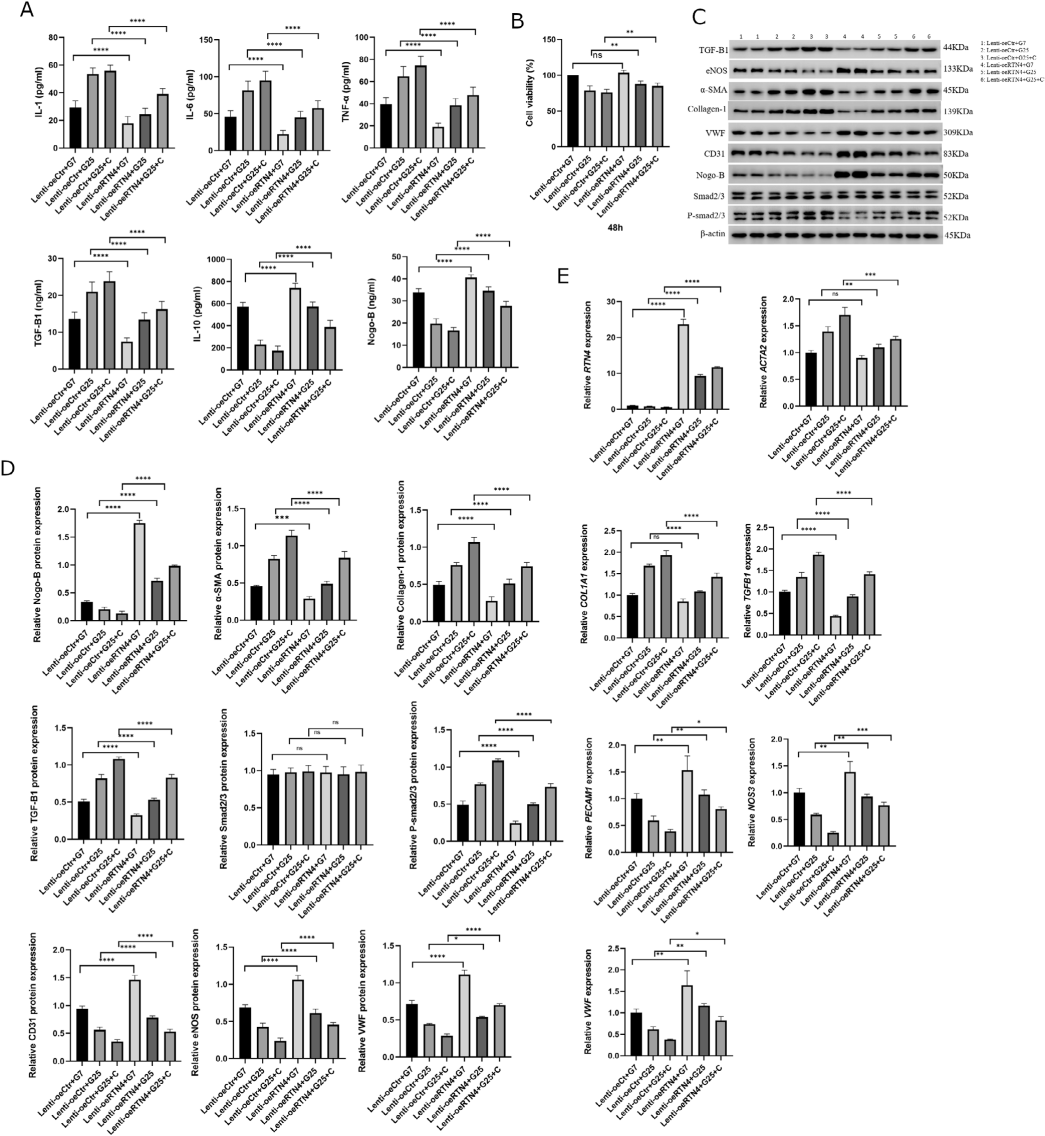
Nogo‐B overexpression alleviated endothelial cell injury. (A) Nogo‐B Overexpression reduced the levels of inflammatory cytokines and TGF‐β1 while increasing the levels of IL‐10 and Nogo‐B, in the cell culture supernatant of HUVECs treated with HG and/or C. (B) Nogo‐B overexpression improved cell viability of HUVECS under hyperglycemic condition. (C, D) Nogo‐B overexpression reduced the expression of mesenchymal marker proteins, TGF‐β1 and P‐smad2/3 while increasing the expression of endothelial marker proteins and Nogo‐B, in HUVECs treated with HG and/or C. (E) Nogo‐B overexpression reduced the expression of mesenchymal marker genes and *TGFB1* while increasing the expression of endothelial marker genes and *RTN4*, in HUVECs under hyperglycemic state. Data are expressed as *M* ± SD. ****p < 0.0001, **p < 0.01, *p < 0.05, ^ns^ *p* > 0.05.

Nogo‐B overexpression was tested by WB (Figure 5). Nogo‐B overexpression reduced inflammatory markers and TGF‐B1 in the cell culture supernatant of HUVECs treated with HG and/or C (Figure 10A). On the contrary, the levels of Nogo‐B and IL‐10 showed a significant increase (Figure 10A). Furthermore, Nogo‐B overexpression improved the cell viability (Figure 10B), tube formation (Figure 7B) and cell migration (Figure 8B) in HUVECs treated with HG and/or C. Interestingly, EndMT was modulated. In fact, mesenchymal markers decreased while endothelial markers increased (Figures 9C,D and 10C–E).

### 5.10 Nogo‐B Knockdown Upregulated TGF‐B Signalling; While Its Overexpression Downregulated TGF‐B It in HUVEC Treated With HG and/or C

Smad2 and Smad3 are the two major downstream regulators that promote TGF‐β1‐mediated tissue fibrosis [16, 40]. Nogo‐B knockdown further increased the expression of TGF‐B1 and P‐smad2/3 (Figure 6C–E), whereas its overexpression reduced the expression of TGF‐B1 (*TGFB1*) and P‐smad2/3 in HUVECs treated with HG and/or C (Figure 10C–E).

## 6 Discussion

This study has shown that T2DM patients with VC (DM + VC) have significantly lower levels of circulating Nogo‐B compared with T2DM patients without VC (DM) or subjects without diabetes (NC). The same trend was observed when Nogo‐B levels of DM were compared with those of NC. IHC analyses also showed that subcutaneous tissues from the feet of T2DM patients with DFU displayed a decrease in the expression of Nogo‐B. To further confirm our findings in humans, HUVECs were treated with HG and/or C. The levels of Nogo‐B decreased in the cell culture supernatant of HUVECs treated with HG and/or C. Moreover, WB and qPCR have shown that the expression of Nogo‐B (*RTN4*) decreased in HUVECs treated with HG and/or C. Our study also demonstrated that Nogo‐B alleviated endothelial cell injury by affecting TGF‐β signalling in a hyperglycemic HUVEC model.

Yang et al. [12] found that Nogo‐B levels increased in plasma of patients with DR. Their findings are inconsistent with ours. In fact, Nogo‐B levels decreased in patients with VC including retinopathy. Within‐group analyses also strengthened our findings. Indeed, T2DM patients with retinopathy did not show any increase in Nogo‐B levels. The possible reason for this inconsistency in the findings is that patients included in their study were only with type 1 diabetes. However, we also acknowledge that the biological heterogeneity, measurement methods, and disease stage may also explain discrepant findings. Nevertheless, our findings have correlation with recent findings. In fact, Hernandez‐Diaz et al. [9] found that Nogo‐B protects the vasculature in diabetic nephropathy and may represent a novel therapeutic target for diabetic vascular complications. Zhang et al. [41] also proved that Nogo‐B contributes to endoplasmic reticulum (ER) stress‐mediated autophagy and protects endothelial cells in diabetic nephropathy.

In multiple logistic regression analyses, lower levels of circulating Nogo‐B were significantly associated with VC when comparing DM + VC with DM or NC. This association remained significant even after adjustment of traditional risk factors for vasculopathy. This should lead us to conclude that low levels of circulating Nogo‐B are independently associated with VC in T2DM patients.

This study also showed that T2DM patients without VC (DM group) have significantly lower levels of Nogo‐B compared with subjects without diabetes. This association was still significant after adjustment for traditional risk factors associated with T2DM. This decrease of Nogo‐B might be associated with a subclinical disease. In fact, in clinical practice, all T2DM patients without obvious VC exhibit other risk factors and/or subclinical organ damage, when carefully assessed [2]. Thus, missed cases of subclinical VC cannot be ruled out in clinical practice. Moreover, in the PoLA guidelines, patients with diabetes are immediately classified as having either high, very high or extreme cardiovascular risk [2].

Lower levels of Nogo‐B in DM + VC were not exclusively related to macro‐ and/or microvascular complications. In addition, lower levels of Nogo‐B in DM + VC or DM were not merely related to glycaemia. This can lead us to conclude that factors other than vascular disease or diabetes may also contribute to the reduction of Nogo‐B in T2DM.

Adjustment for vascular protective drugs did not change significantly the association of lower levels of Nogo‐B with VC in T2DM patients. The same trend was found after adjustment for cardioprotective drugs and vascular risk factors taken together (*p* < 0.001). This can lead us to conclude that cardioprotective drugs might not affect significantly the association of lower levels of Nogo‐B with vascular disease. On the other hand, adjustment for glucose lowering drugs (combined or used alone) did not affect significantly the association of lower Nogo‐B levels with VC and/or history of DFU (*p* < 0.001). It is important to note that lower levels of Nogo‐B were still associated with vascular disease after a fully adjusted multivariate model (combined adjustment for vascular risk factors and treatment), when comparing DM + VC with NC (*p* < 0.001). The same trend was found after adjustment for all covariates (combined adjustment for vascular risk factors, treatments and HbA1c), when DM + VC was compared with DM.

In DM + VC, DF‐P displayed significantly lower levels of circulating Nogo‐B than DF‐N (*p* < 0.001). In addition, logistic regression analyses showed lower levels of Nogo‐B were associated with history of DFU even after adjustment for factors associated with DFU. The further low levels of Nogo‐B in DF‐P might be due to the severity of vasculopathy in T2DM. In fact, there is a complex link between DFU and cardiac disease even though the mechanism remains poorly understood [42]. Inflammation, endothelial dysfunction, and oxidative stress may contribute to accelerated atherosclerosis, thrombosis, and cardiomyocyte dysfunction in the setting of DFU [42]. Whether accentuated decrease of Nogo‐B in T2DM patients with DFU may represent or predict a more severe form of vasculopathy in T2DM patients still needs further investigation.

We demonstrated that Nogo‐B alleviates endothelial cell injury by inhibiting the TGF‐β signalling pathway in HUVECs treated with HG and/or C. This may correlate with previous findings. In fact, a recent study found that Nogo‐B overexpression attenuated AKT phosphorylation in the kidney cortex lysate of diabetic mice [9]. Another study previously demonstrated that TGF‐β/Smad3 induced AKT phosphorylation in vascular smooth muscle cells [43]. In addition, crosstalk between endothelial cells and vascular smooth muscle cells has been previously shown [44]. This led us to conclude that Nogo‐B may protect vasculature in diabetes through pathways involving canonical and non‐canonical TGF‐β signalling. Further studies should reassess the issue.

Inflammatory cytokines and TGF‐β1 simultaneously increased in the cell culture supernatant of HUVECs treated with HG and/or C. This correlates with previous findings. In fact, studies demonstrated that there is a crosstalk between the NF‐κB signalling pathway and TGF‐β [45]. Indeed, in human corneal epithelial cells, TGF‐β could induce atypical RNA stress responses, leading to accelerated mRNA degradation of IκBα, an inhibitor of NF‐κB [45]. Activated NF‐κB induces an array of gene expressions of inflammatory cytokines [45]. Interestingly, in our study, Nogo‐B overexpression simultaneously reduced the levels of inflammatory cytokines and TGF‐β1 in the cell culture supernatant of HUVECs treated with HG and/or C.

**FIGURE 11:**
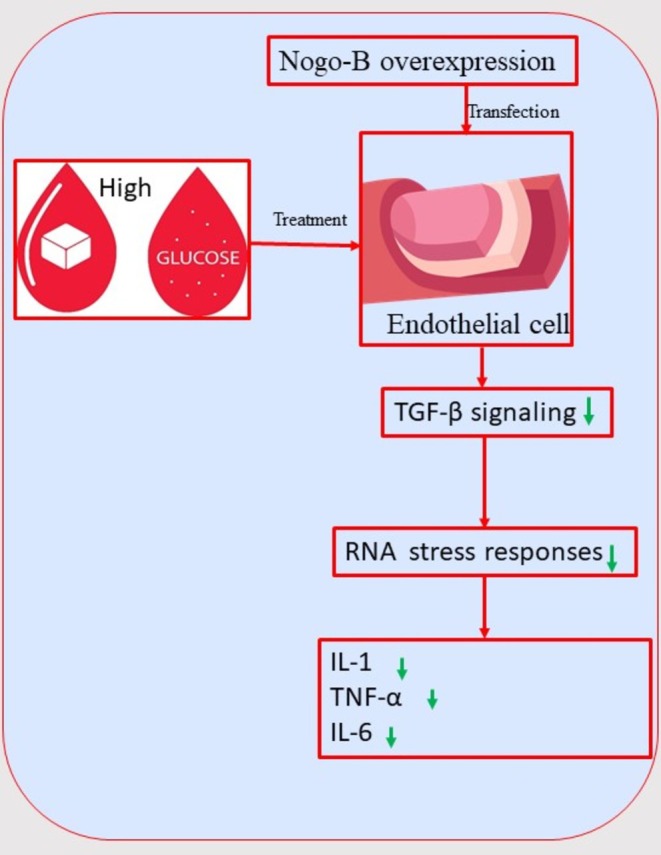
Schematic figure illustrating the mechanism by which Nogo‐B may protect endothelial cells under hyperglycemic state.

Mechanistically, Nogo‐B may inhibit TGF‐β signalling, which in turn will lead to the decrease in RNA stress responses. The decrease in RNA stress responses may ultimately reduce inflammatory cytokines due to the increase of IκBα (schematic figure: Figure 11). This may correlate in part with previous findings. In fact, previous results showed that the expression of Nogo‐B decreased in M1 macrophages but increased in M2 macrophages in bone marrow derived macrophages [46]. M2 macrophages release anti‐inflammatory enzymes and cytokines such as CD163, IL‐10, arginase‐1 (Arg‐1), scavenging receptor and mannose receptor (MR) [46].

By hyperglycemic HUVEC model, our study proved that Nogo‐B alleviated endothelial cell injury. In fact, previous studies demonstrated that Nogo‐B influences key functions in vascular smooth muscle cells by modulating microtubules and actin organisation/dynamics via Rho A activation and HDAC‐6 expression and activity [47]. Furthermore, they also proved that Nogo‐B coordinates macrophage‐mediated inflammation with arteriogenesis, wound healing, and blood flow control [48].

The study has some limitations. First, the largely retrospective nature of this clinical study is a limitation. Second, the DM and DM + VC groups are unbalanced. In fact, most of the patients recruited had at least one macro‐or microangiopathy. The main cause is that our institution is a tertiary hospital and most of our patients are those with long standing diabetes. Nevertheless, when reducing the sample sizes of DM + VC and/or NC to the level of DM, the same trends of results were found (*p* < 0.001). Third, in the in vitro study, although Nogo‐B and TGF‐β signalling behave inversely, other explanations are possible, like indirect effects or feedback loops. TGF‐β signalling might be a response to changes in Nogo‐B rather than a direct result. To prove cause‐and‐effect, experiments such as inhibition or rescue experiments were needed. However, due to a lack of enough materials, these experiments were not performed. Fourth, in vitro experiments test only a short term of high‐glucose exposure, whereas diabetes is a long‐term disease. We do not know whether long‐term changes in Nogo‐B levels in the body would have the same effects as the short‐term cell experiments. HUVECs are a good model for human endothelial cells. However, they do not fully recapitulate the complex environment of the human body. They lack interaction with other cell types, blood flow, and whole‐body factors. Fifth, different ELISA kits may produce different results. Last, our study still lacks an in vivo study to support our evidence.

Nevertheless, our study presents some strengths. In fact, it has included a relatively large number of patients with T2DM, which lacks in previous studies. Indeed, previous studies on Nogo‐B and vascular diseases in diabetes were largely limited to models (in vivo and/or in vitro) of type 1 diabetes or microvascular diseases of diabetes [9, 12, 41].

## 7 Conclusion

This study has shown that low levels of Nogo‐B are associated with vascular disease in T2DM patients. Furthermore, patients with DFU history displayed further low levels of Nogo‐B. Our findings have also shown that Nogo‐B alleviates endothelial cell injury by affecting TGF‐β signalling, though direct evidence is lacking. Nogo‐B might be a promising target for the treatment and/or diagnosis of vascular diseases in diabetes. However, due to limitations of this study, further studies are still needed to verify or support our findings.

## Author Contributions

L.I. and Q.C. conceived and designed the study. X.L., L.I. and X.C. analysed the data. All authors interpreted the data. L.I., Y.G. and X.L. collected the samples. L.I. performed all cell culture related works. L.M. contributed to cell culture related works by providing fruitful advices. L.I. and X.X. wrote the first draft of the manuscript. All authors participated in its critical review with important intellectual contributions, and approved the final version of the manuscript. X.X. and Q.C. are the guarantors of this work and, as such, had full access to all the data in the study and take responsibility for the integrity of the data and the accuracy of the data analysis.

## Funding

This work was supported by National Natural Science Foundation of China (81871222 and 81570763).

## Ethics Statement

The Ethics Committee of the First Affiliated Hospital of Chongqing Medical University approved this study (Number: K2023‐537). The experimental procedures were conducted according to the Declaration of Helsinki.

## Consent

All authors have seen and approved the final version of the manuscript being submitted.

## Conflicts of Interest

The authors declare no conflicts of interest.

## Data Availability

The datasets used and/or analysed for this study are available from the corresponding authors upon reasonable request.
